# Effect of timing on patient-reported outcomes in contralateral symmetrization surgery in autologous breast reconstruction

**DOI:** 10.1093/bjsopen/zrag023

**Published:** 2026-05-13

**Authors:** Rojda Gümüscü, Olivia Sjökvist, Ellen Kragsterman, Johan Svensson, Susanna Kauhanen, Maria Mani, Rebecca Wiberg

**Affiliations:** Department of Surgical Sciences, Faculty of Medicine, Uppsala University, Uppsala, Sweden; Department of Plastic and Maxillofacial Surgery, Uppsala University Hospital, Uppsala, Sweden; Department of Surgical Sciences, Faculty of Medicine, Uppsala University, Uppsala, Sweden; Department of Plastic and Maxillofacial Surgery, Uppsala University Hospital, Uppsala, Sweden; Department of Plastic and Maxillofacial Surgery, Uppsala University Hospital, Uppsala, Sweden; Department of Statistics, Umeå School of Business, Economics and Statistics, Umeå University, Umeå, Sweden; Department of Surgical Sciences, Faculty of Medicine, Uppsala University, Uppsala, Sweden; Department of Plastic Surgery, University of Helsinki and Helsinki University Hospital, Helsinki, Finland; Department of Surgical Sciences, Faculty of Medicine, Uppsala University, Uppsala, Sweden; Department of Plastic and Maxillofacial Surgery, Uppsala University Hospital, Uppsala, Sweden; Department of Diagnostics and Intervention, Plastic Surgery and Surgery, Umeå University, Umeå, Sweden

**Keywords:** BREAST-Q™, contralateral breast surgery, patient satisfaction

## Abstract

**Background:**

The optimal timing of contralateral symmetrization surgery (SS) in unilateral autologous breast reconstruction remains under debate. This study compared short- and long-term patient-reported outcomes between women undergoing contralateral SS during the index breast reconstruction surgery and those undergoing contralateral SS as a separate secondary surgery.

**Methods:**

A quantitative study evaluating clinical and patient-reported outcomes was conducted using prospectively collected BREAST-Q™ questionnaires and retrospectively collected patient data. All patients undergoing unilateral abdominal-based free flap breast reconstruction at Uppsala University Hospital between January 2016 and December 2023 were considered for study participation. Those who had undergone contralateral SS or were scheduled for SS on the contralateral breast at a later date and who had completed preoperative and/or postoperative patient surveys were included in the study.

**Results:**

In all, 91 patients were included in the study. Index breast reconstruction was performed immediately at the time of mastectomy in seven patients (7.7%) and as delayed reconstruction in 84 patients (92.3%). Forty-six patients (50.5%) underwent simultaneous contralateral SS and 45 patients (49.5%) underwent secondary contralateral SS. Among women undergoing delayed breast reconstruction, the difference in satisfaction with breasts at baseline and 3 months after the index reconstruction, was significantly higher among those undergoing simultaneous contralateral SS than among those waiting for a secondary contralateral SS (*P* = 0.011). Two years after the index reconstruction, when all patients were symmetrized, there was no difference in satisfaction with breasts between the two groups. There was a moderate increase in operation time at index breast reconstruction between the simultaneous and secondary contralateral SS groups (mean(s.d.) 371.8(79.1) *versus* 337.6(61.0) minutes, respectively; *P* = 0.036). There was no significant difference in the overall complication rate between the two groups (*P* = 0.094). As expected, the number of additional operations under general anaesthesia following the index breast reconstruction was significantly higher in the secondary contralateral SS group (*P* = 0.007).

**Conclusion:**

Simultaneous contralateral SS may increase short-term satisfaction with breasts and lead to fewer additional surgeries requiring general anaesthesia. However, in the long term (2-year follow-up), patient satisfaction with breasts was not affected by the timing of the contralateral SS.

## Teaser

This cohort study compared patient-reported outcomes between simultaneous and secondary contralateral breast symmetrization in women undergoing unilateral autologous breast reconstruction. Short-term satisfaction was better with simultaneous symmetrization; however, once all patients had undergone symmetrization, the difference in satisfaction was no longer significant.

## Introduction

Abdominal-based free flaps are a versatile and widely used technique in post-mastectomy breast reconstruction because they enable natural and long-lasting results. The primary goals of breast reconstruction are to restore the physical form of the resected breast and to recreate chest wall balance and symmetry, which plays a significant role in patient perceived wellbeing and overall quality of life^1–4^.

For patients with unilateral breast cancer necessitating mastectomy, management of the contralateral breast is a key consideration in achieving chest wall symmetry, with several factors influencing the decision to proceed with contralateral symmetrization surgery (SS). These factors include native breast size, shape, breast ptosis, tissue availability, distribution of the donor site, and, crucially, patient preferences and expectations. Furthermore, when relevant, practical issues such as theatre and surgeon availability, insurance coverage, patient co-morbidities, and surgical timing relative to adjuvant therapies need to be taken into consideration. These factors should be considered during the preoperative consultation to allow for shared decision-making with the patient and individually tailored reconstructive surgery to optimize patient satisfaction and health-related quality of life (HRQoL)^5,6^.

The practice of contralateral SS is well established in unilateral breast reconstruction^7–9^. The timing of the procedure, either simultaneously with the index breast reconstruction or as a separate secondary procedure, is often subjective and remains a topic of debate. Previous studies on autologous breast reconstruction have shown that simultaneous contralateral SS does not increase the risk of postoperative complications^10–14^ and likely does not prolong the operative time during index breast reconstruction surgery^15,16^.

Measuring patient-reported outcomes (PROs), including satisfaction and function, is an integral component in evaluating breast reconstruction^17,18^. However, literature specifically addressing PROs in relation to the timing of contralateral SS is lacking.

Thus, the aim of the present study was to compare PROs between women undergoing simultaneous contralateral SS and those undergoing secondary contralateral SS after abdominal-based free flap breast reconstruction.

## Methods

### Study design and patient population

In this quantitative study, data were collected retrospectively from electronic patient records and data on PROs were collected prospectively: as part of routine clinical practice, all patients undergoing breast reconstruction were invited to respond to patient surveys before and at predetermined intervals after surgery.

All women who underwent unilateral abdominal-based free flap breast reconstruction at the Plastic Surgery Department at Uppsala University Hospital between January 2016 and December 2023 were considered for the study. Those who had undergone contralateral SS, either at the time of breast reconstruction or as a secondary procedure, or where waiting for contralateral SS as a secondary procedure, and who had completed preoperative and/or postoperative patient surveys were included in this study. SS was defined as surgery that changed the form or volume of the contralateral breast, and included mastopexy, breast reduction, liposuction or lipofilling, and implant augmentation. Patients with revisional SS limited to the ipsilateral reconstructed breast, those in whom contralateral SS was performed before breast reconstruction, and those who experienced free flap loss at their index breast reconstruction were excluded from the study. Patients undergoing simultaneous bilateral autologous breast reconstruction, such as those with bilateral breast cancer or pathogenic gene variants, were also excluded from the study. Contralateral mastectomy with reconstruction was not offered for symmetrization purposes alone at the study institution.

Local institutional criteria for abdominal-based free flap breast reconstruction were a body mass index (BMI) ≤30 kg/m^2^ and abstinence from nicotine for 6 weeks before and for 6 weeks after surgery. Breast reconstruction was performed both in the delayed and immediate setting following mastectomy. Preoperative abdominal computed tomography angiograms for perforator mapping were performed in all patients. A contralateral mammogram was required in all patients within 3 months before SS. The decision to offer contralateral SS was at the discretion of the lead surgeon, with different preferences regarding timing among surgeons.

The primary outcome of this study was patient-reported satisfaction. Secondary outcomes were the rate of complications and the rate of additional surgery.

### Data collection

The cohort was established through retrospective review of a prospectively maintained database of all breast reconstructions performed at Uppsala University Hospital from 2016 onwards. In January 2016, patient surveys were introduced into routine clinical practice and patients were invited to respond to these surveys before surgery and 3 months and 2 years after the index breast reconstruction. Electronic patient records were reviewed and patient data were collected using a digital spreadsheet. The data collected included patient characteristics (age, BMI at index surgery, co-morbidities); clinical assessment of breast volume and form; the date of surgery and operative details (surgery performed, laterality, operative time, flap weight, and, in the case of immediate breast reconstruction, mastectomy weight); details pertaining to contralateral SS (technique used, weight of resected specimen, volume of fat graft or liposuction); postoperative complications; and any subsequent additional surgery performed to complete breast reconstruction. Complications occurring within 30 days of surgery are reported descriptively and were classified by the subsequent intervention as per the Clavien–Dindo (CD) system^19^.

### Patient-reported outcomes

All patients were asked to complete patient surveys before or during their first visit to the plastic surgery outpatient department according to standard clinical routines. Follow-up questionnaires were sent by letter with a reply-paid envelope 3 months and 2 years after the index breast reconstruction. For the present study, the BREAST-Q™ Reconstructive Module (version 1.0)^20^ was used to assess HRQoL, shown in *supplementary methods*. The BREAST-Q™ questionnaire was chosen because it has been extensively validated, is procedure-specific, and designed to assess PRO measures in breast surgery patients^21–23^. The BREAST-Q™ assesses four quality of life scales (physical wellbeing chest, physical wellbeing abdomen, psychosocial wellbeing, and sexual wellbeing) and five satisfaction scales (breasts, medical team, information, office staff, and surgeon). The satisfaction with breasts item has four questions and patients are asked to grade their satisfaction on a four-point scale. A raw score is calculated from the sum of each individual scale. The raw scores for each module are transformed according to the scoring tables provided by the BREAST-Q™ team. The transformed scores range from 0 to 100, with a higher score indicating either greater satisfaction or wellbeing. A minimum difference score of 4 in the transformed scores has been suggested to be clinically useful when using the BREAST-Q™ reconstructive module^24^.

### Ethical considerations

This study was performed according to the principles of the Declaration of Helsinki and the ethical guidelines of the Swedish Research Council. Written informed consent was obtained from all patients. Ethics approval for the study was obtained from the national Swedish Ethical Review Board (Diarienummer 2014-354 and 2021-05146).

### Statistical analysis

Study population characteristics were calculated as means and percentages. BREAST-Q™ scores and differences between baseline and later scores were modelled as continuous variables. Comparisons were made between the simultaneous and secondary contralateral SS groups using Fisher's exact test or the Fisher–Freeman–Halton exact test extension for categorical variables, and independent-sample *t* tests for continuous variables. Missing data in *Table 1* relating to patient characteristics were assumed to be missing at random. All statistical tests were two sided and significance was set at *P* < 0.05. Statistical analyses were performed using IBM^®^ SPSS^®^ Statistics software version 29.0.0 (IBM, Armonk, NY, USA). In the secondary contralateral SS cohort, four patients were candidates for contralateral SS when they responded to their 3-month patient survey, but the surgery had not been conducted at the time of the 2-year follow-up (one patient had recurrent breast cancer, one patient was lost to follow-up after moving abroad, one patient was found to have a pathogenic gene variant and underwent contralateral risk reducing mastectomy, and the surgery was postponed in one patient because of personal preferences). The seven patients undergoing immediate breast reconstruction were excluded from comparative analyses pertaining to PRO measures to ensure homogeneity in the study population.

**Table 1:** Characteristics of the study cohort divided by timing of the contralateral symmetrization surgery (SS), simultaneous to index breast reconstruction or as a secondary surgery

	All (*n* = 91)	Simultaneous SS (*n* = 46)	Secondary SS (*n* = 45)	P*
Age, mean(s.d.)	53.8(8.3)	55.5(8.6)	51.9(7.7)	0.036
BMI (kg/m^2^), mean(s.d.)	26.7(2.5)	26.6(2.5)	26.9(2.6)	0.601
**Smoking status**				0.396
Non-smoker	51 (58.0%)	24 (53.3%)	27 (62.8%)	
Smoking cessation > 6 weeks before surgery	37 (42.0%)	21 (46.7%)	16 (37.2%)	
Hypertension	22 (24.2%)	10 (21.7%)	12 (26.7%)	0.631
Diabetes	3 (3.3%)	2 (4.3%)	1 (2.2%)	1.000
Depression/anxiety	14 (15.4%)	6 (13.0%)	8 (17.8%)	0.574
**Timing of reconstruction**				0.267
Immediate	7 (7.7%)	2 (4.3%)	5 (11.1%)	
Delayed	84 (92.3%)	44 (95.7%)	40 (88.9%)	
**Duration of index surgery (min), mean(s.d.)**				
Immediate breast reconstruction	350.1(160.1)	291.5(16.3)	373.6(189.7)	0.588
Delayed breast reconstruction	356.0(72.9)	371.8(79.1)	337.6(61.0)	0.036
**Symmetrization technique**				0.127
Reduction	53 (60.9%)	32 (69.6%)	21 (51.2%)	
Mastopexy	28 (32.2%)	13 (28.3%)	15 (36.6%)	
Fat grafting	2 (2.3%)	1 (2.2%)	1 (2.4%)	
Combination	3 (3.4%)	0 (0.0%)	3 (7.3%)	
Implant augmentation	1 (1.1%)	0 (0.0%)	1 (2.4%)	
Missing data	4	0	4†	

Values are *n* (%) unless specified otherwise. *P-values were generated using Fischer's exact test for categorical variables and independent-sample *t* tests for continuous variables. †Four patients in the secondary cohort were candidates for contralateral SS but surgery had not yet been conducted for various reasons, as detailed in the Methods section. SS, symmetrization surgery; s.d., standard deviation; BMI, body mass index; min, minutes.

## Results

### Study population

In all, 91 patients met the selection criteria and were included in the study. Of these 91 patients, 46 (50.5%) and 45 (49.5%) underwent simultaneous and secondary contralateral SS respectively. Seven patients (7.7%) had immediate and 84 patients (92.3%) had delayed index breast reconstruction. Patient characteristics are presented in *Table 1*. The patients in the simultaneous contralateral SS cohort were older than those in the secondary contralateral SS cohort (mean age 55.5 *versus* 51.9 years; *P* = 0.036). There were no significant differences in BMI, smoking status, or co-morbidities (hypertension, diabetes, and depression/anxiety) between the two groups. The free flap breast reconstruction was based on deep inferior epigastric vessels in 90 patients (98.9%) and on superficial inferior epigastric vessels in 1 patient (1.1%). The most common method of contralateral SS in the simultaneous and secondary contralateral SS groups was breast reduction (32 (69.6%) *versus* 21 (51.2%), respectively), followed by mastopexy (13 (28.3%) *versus* 15 (36.6%), respectively). One patient in each cohort underwent fat grafting alone. One patient had contralateral implant augmentation. The mean(standard deviation (s.d.)) time from index breast reconstruction to contralateral SS in the secondary contralateral SS group and to the first additional surgery in the simultaneous contralateral SS group was 13.4(6.3) and 13.3(8.8) months, respectively. The mean difference in operative time for the immediate index breast reconstruction did not differ between the two groups, but in the case of delayed index breast reconstruction, the difference between the simultaneous and secondary contralateral SS groups was 34.2 minutes (371.8 *versus* 337.6 minutes, respectively; *P* = 0.036).

### BREAST-Q™ questionnaire

At baseline, 42 (91%) and 41 (91%) patients in the simultaneous and secondary contralateral SS groups, respectively, responded to the BREAST-Q™ questionnaire. The response rate declined at both postoperative follow-up timepoints and there was a difference in response rates for the different subscales. The response rates to the BREAST-Q™ questionnaire in the simultaneous and secondary contralateral SS groups were 65% (30 patients) and 64% (29), respectively, at 3 months and 48% (22) and 58% (26), respectively, at 2 years.

The mean(s.d.) BREAST-Q™ scores for satisfaction with breasts after immediate breast reconstruction were 58(9) and 38(24) in the simultaneous (2 patients) and secondary (5) contralateral SS groups, respectively, at baseline and 68(28) in the secondary cohort 2 years after the index breast reconstruction (no BREAST-Q™ scores were available at the 2-year follow-up for the simultaneous contralateral SS group). The immediate breast reconstruction group was too small to analyse further and is not included in the comparative analysis below.

**Fig. 1:**
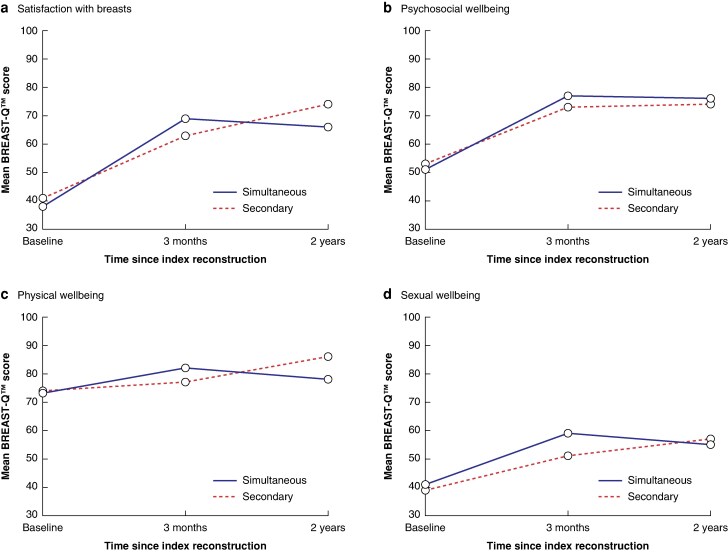
Mean BREAST-Q™ scores at baseline and 3 months and 2 years after the index breast reconstruction **a** Satisfaction with breasts and **b** psychosocial, **c** physical, and **d** sexual wellbeing, as determined using the BREAST-Q™, at baseline and 3 months and 2 years after the index breast reconstruction following simultaneous and secondary contralateral SS. In the secondary contralateral SS cohort, all women were waiting for symmetrization surgery at the 3-month follow-up. All women had subsequently undergone symmetrization surgery 2 years after the index breast reconstruction. The seven women who underwent mastectomy with immediate index breast reconstruction were excluded from this analysis.

There was no significant difference in the mean(s.d.) baseline BREAST-Q™ scores for satisfaction with breasts after delayed index breast reconstruction between the simultaneous and secondary contralateral SS groups (38(17) *versus* 41(17), respectively; *P* = 0.445). The preoperative mean values for the psychosocial, physical, and sexual wellbeing subscales did not differ significantly between the two groups. The mean BREAST-Q™ scores are presented in *Fig. 1* and *Table S1*. When looking at the difference in satisfaction with breasts between baseline and 3 months after the index breast reconstruction, there was a significant difference between the two groups, with women undergoing simultaneous contralateral SS having a higher difference in BREAST-Q™ scores than women waiting for a contralateral SS (*P* = 0.011; *Table 2*). At 2 years after the index breast reconstruction, once all women had been operated on on the contralateral side, there was no significant difference in the mean difference in satisfaction with breasts between the two groups (3.0; 95% confidence interval (c.i.) −13.5 to 19.5; *P* = 0.714).

**Table 2:** Difference in mean BREAST-Q™ scores between baseline and 3 months and 2 years after the index breast reconstruction, according to timing of symmetrization surgery

	BREAST-Q™ scores, mean(s.d.), *n*		Mean difference simultaneous *versus* secondary SS*	*P*
	Simultaneous SS	Secondary SS		
**Difference in satisfaction with breasts**				
Baseline *versus* 3 months after surgery†	34.8(26.1), 27	17.7(19.9), 25	17.1 (4.1, 30.1)	0.011
Baseline *versus* 2 years after surgery‡	32.2(27.7), 21	29.2(19.4), 16	3.0 (−13.5, 19.5)	0.714
**Difference in psychosocial wellbeing**				
Baseline *versus* 3 months after surgery†	27.0(23.2), 27	17.3(15.0), 23	9.6 (−1.7, 20.9)	0.094
Baseline *versus* 2 years after surgery‡	24.0(22.3), 21	16.6(22.6), 16	7.4 (−7.7, 22.5)	0.325
**Difference in physical wellbeing**				
Baseline *versus* 3 months after surgery†	8.6(13.1), 27	1.2(14.7), 24	7.5 (−0.3, 15.3)	0.060
Baseline *versus* 2 years after surgery‡	4.9(15.8), 19	8.8(10.4), 16	−3.9 (−13.3, 5.5)	0.402
**Difference in sexual wellbeing**				
Baseline *versus* 3 months after surgery†	16.3(22.0), 16	7.8(16.1), 22	8.5 (−4.1, 21.0)	0.179
Baseline *versus* 2 year after surgery‡	17.9(17.5), 14	12.3(20.3), 12	5.7 (−9.6, 21.0)	0.451

*Values in parentheses are 95% confidence intervals. The seven women who underwent immediate breast reconstruction were excluded from this analysis. †In the secondary cohort, all women were waiting for contralateral symmetrization surgery at the 3-month follow-up. ‡All women had undergone contralateral symmetrization surgery 2 years after the index surgery. s.d., standard deviation; SS, symmetrization surgery.

The mean difference in psychosocial, physical, or sexual wellbeing did not differ significantly between the two groups at either 3 months or 2 years after the index breast reconstruction. Furthermore in the subgroup analysis on patients with contralateral breast reduction (53 patients; all other SS techniques excluded), the results mirrored those above (data not shown): women undergoing simultaneous breast reduction had a significantly higher difference in satisfaction with breasts 3 months after the index breast reconstruction compared with baseline than women waiting for a contralateral breast reduction (23.1; 95% c.i. 3.7 to 42.5; *P* = 0.021), whereas this difference was not observed at 2 years (4.6; 95% c.i. −18.6 to 27.8; *P* = 0.685).

The mean(s.d.) BREAST-Q™ scores for satisfaction with outcome did not differ between the simultaneous and secondary contralateral SS groups at 2 years after the index autologous breast reconstruction (79.6(27.3) *versus* 79.4(18.5), respectively; *P* = 0.971). Similarly, there was no significant difference in the scores for the stand-alone question ‘How your reconstructed breast(s) look now compared to before you had any breast surgery?’ between the simultaneous and secondary contralateral SS groups (mean(s.d.) 3.3(0.8) *versus* 3.6(0.6), respectively; *P* = 0.195).

### Postoperative complications

Among all 91 patients, 42 (46.2%) were recorded as experiencing a postoperative complication requiring intervention within 30 days of the index breast reconstruction. These complications are summarized in *Table 3*. The simultaneous and secondary contralateral SS groups had similar rates of overall complications for the reconstructed breast (9 *versus* 17, respectively; *P* = 0.066) and abdominal donor site (6 *versus* 12, respectively; *P* = 0.121). Of all 42 patients with complications, 35 (83.3%) had complications classed as CD grade I or II. Eight patients (2 and 6 in the simultaneous and secondary contralateral SS groups, respectively) required reoperation for complications (CD IIIb) to the reconstructed breast: four patients had vascular compromise, one patient had a haematoma, and two patients developed fat necrosis that was excised in the operating theatre as a day surgery procedure. One patient had an abdominal drain suture that was removed under local anaesthesia (CD IIIa). Of the 46 patients who underwent simultaneous contralateral SS, four had local infections to the symmetrized breast requiring oral antibiotics (CD II).

**Table 3:** Rate of complications within 30 days of index breast reconstruction overall and according to the timing of the intervention

	All (*n* = 91)	Simultaneous SS (*n* = 46)	Secondary SS (*n* = 45)	P*
Any complication	42 (46.2%)	17 (37.0%)	25 (55.6%)	0.094
**Flap complications**				0.066
CD grade I	3 (3.3%)	3 (6.5%)	0 (0%)	
CD grade II	15 (16.5%)	4 (8.7%)	11 (24.4%)	
CD grade IIIb	8 (8.8%)	2 (4.3%)	6 (13.3%)	
**Donor site complications**				0.121
CD grade I	5 (5.5%)	1 (2.2%)	4 (8.9%)	
CD grade II	12 (13.2%)	5 (10.9%)	7 (15.6%)	
CD grade IIIa	1 (1.1%)	0 (0%)	1 (2.2%)	
**Complication to symmetrized breast**				
CD grade II	4 (4.4%)	4 (8.7%)	–	

Values are *n* (%) unless stated otherwise. Complications were graded using the CD classification system^19^ and according to operative site. *P values were generated using Fisher’s exact test. SS, symmetrization surgery; CD, Clavien–Dindo.

### Additional surgery

For ease of data interpretation, each additional surgery was categorized by anatomical site (flap, contralateral breast, nipple alone, abdomen), as presented in *Table 4*. Examples of procedures performed include mastopexy, breast reduction, liposuction, lipofilling, scar correction, and nipple reconstruction.

**Table 4:** Additional surgery after index breast reconstruction

	All (*n* = 77)	Simultaneous SS (*n* = 36)	Secondary SS (*n* = 41)	P*
Total no. of additional surgeries, mean(s.d.)	1.35(0.56)	1.36(0.64)	1.34(0.48)	0.878
Total no. of additional surgeries under general anaesthesia, mean(s.d.), *n*†	0.94(0.63), 71	0.74(0.74), 35	1.14(0.42), 36	0.007
**First additional surgery**	76 (98.7%)	35 (97.2%)	41 (100%)	0.468
No further surgery‡	1 (1.3%)	1 (2.8%)		
Type of surgery				<0.001
Surgery to contralateral breast	10 (13.2%)	0 (0.0%)	10 (24.4%)	
Surgery to flap	11 (14.5%)	11 (31.4%)	0 (0.0%)	
Surgery to both flap and contralateral breast	45 (59.2%)	14 (40.0%)	31 (75.6%)	
Nipple reconstruction only	11 (13.2%)	10 (28.6%)	0 (0.0%)	
Type of anaesthesia				<0.001
General anaesthesia	52 (73.2%)	18 (51.4%)	34 (94.4%)	
Local anaesthesia	19 (26.8%)	17 (48.6%)	2 (5.6%)	
**Second additional surgery**	26 (33.8%)	12 (33.3%)	14 (34.1%)	1.000
Type of surgery				0.122
Surgery to contralateral breast	3 (11.5%)	3 (25.0%)	0 (0.0%)	
Surgery to flap	6 (23.1%)	4 (33.3%)	2 (14.3%)	
Surgery to both flap and contralateral breast	5 (19.2%)	1 (8.3%)	4 (28.6%)	
Nipple reconstruction only	11 (42.3%)	4 (33.3%)	7 (50.0%)	
Abdominal correction only	1 (3.8%)	0 (0.0%)	1 (7.1%)	
Type of anaesthesia				1.000
General anaesthesia	13 (50.0%)	6 (50.0%)	7 (50.0%)	
Local anaesthesia	13 (50.0%)	6 (50.0%)	7 (50.0%)	
**Third additional surgery**	2 (2.6%)	2 (5.6%)	0 (0.0%)	0.221
Type of surgery				–
Surgery to contralateral breast	2 (100.0%)	2 (100.0%)	0 (0.0%)	
Type of anaesthesia				
General anaesthesia	2 (100.0%)	2 (100.0%)	0 (0.0%)	

Values are *n* (%) unless stated otherwise. The total number of women included in this analysis was 77; 14 patients were excluded, ten of whom were in the simultaneous contralateral SS group (five awaiting additional surgery, five re-referred to their home hospital) and four were in the secondary contralteral SS group (candidates for contralateral SS who had not yet undergone the procedure due to reasons stated in the Methods section). †The number of women here differs from the total number of women included in the analysis due to missing data for five in the secondary contralateral SS group and one in the simultaneous SS (women with no further surgery) group. ‡One patient in the simultaneous contralateral SS group had no additional surgery due to patient choice. *P values were generated using Fisher’s exact test. SS, symmetrization surgery; s.d., standard deviation.

The mean number of additional surgeries after the index breast reconstruction was comparable between the simultaneous and secondary contralateral SS groups (mean(s.d.) 1.36(0.64) *versus* 1.34(0.48), respectively; *P* = 0.878). In all, 35 of 36 patients (97.2%) underwent additional surgery following simultaneous contralateral SS (one woman chose to defer surgery while on the waiting list), with surgery to both flap and contralateral breast being the most common (14 of 35, 40%). Of the 36 patients who underwent additional surgery, 12 (33.3%) had a second additional surgery and 2 (5.6%) had a third additional surgery. In the secondary contralateral SS group, 31 of 41 patients (75.6%) had surgery to both the flap and contralateral breast and 10 (24.4%) had surgery to the contralateral breast alone at the first surgery (that is, SS) after index breast reconstruction. Furthermore, of these 41 patients, 14 (34.1%) had a second additional surgery following their symmetrization, of which 7 (50.0%) were nipple reconstructions alone and 1 (7.1%) was an abdominal correction alone. The mean(s.d.) number of additional surgeries after the index breast reconstruction performed under general anaesthesia was greater for the secondary contralateral SS group than the simultaneous contralateral SS group (1.14(0.42) *versus* 0.74(0.74); *P* = 0.007). Overall, breast reconstruction was completed under local anaesthesia only in 14 of 35 patients (40%) in the simultaneous contralateral SS group.

## Discussion

Decisions regarding the timing of contralateral SS in unilateral abdominal-based free flap breast reconstruction are multifaceted and vary across institutions, with decision-making approaches ranging from individual surgeon preferences to guidance through multidisciplinary team discussions. The findings of the present study show that performing contralateral SS at the time of index breast reconstruction led to higher levels of satisfaction with breasts in the early postoperative period and reduced the overall number of additional surgeries under general anaesthesia to complete breast reconstruction. However, at the 2-year follow-up, once all women were symmetrized, patient-reported satisfaction with breasts was equivalent between the simultaneous and secondary contralateral SS groups.

Unsurprisingly, patients with secondary symmetrization required more surgeries under general anaesthesia to complete breast reconstruction (mean(s.d.) 1.14(0.42) *versus* 0.74(0.74); *P* = 0.007). These findings mirror those reported by Peltristo *et al*.^14^, who found a reduced total number of surgical procedures and subsequently a shorter reconstructive journey with simultaneous *versus* secondary SS in women having autologous breast reconstruction. Collectively, these findings suggest advantages in both early patient satisfaction and overall resource use in performing simultaneous SS. This can be especially significant in healthcare systems with longer waiting times for secondary surgery.

Perceived challenges that may prevent surgeons from offering simultaneous contralateral SS include an assumption that adding contralateral surgery would prolong operative time at the index breast reconstruction and increase complication rates, or that delaying contralateral surgery leads to a more symmetrical result because the flap has settled. Furthermore, some patients may be undecided about undergoing surgery to their contralateral breast at the time of index breast reconstruction, reflecting patient preferences. Previous studies examining the timing of contralateral SS in deep inferior epigastric perforator flap breast reconstruction have shown either a modest increase or no difference in operative time at index breast reconstruction^11,14–16^. The overall rate of complications at the index breast reconstruction does not seem to differ if the contralateral SS was performed simultaneously or not^11,15,16^. The findings in the present study align with those of previous studies, indicating that contralateral SS at the time of the index breast reconstruction is associated with a modest increase in operative time, without a significant difference in the overall rate of complications. The modest increase in operative time at the index breast reconstruction for simultaneous contralateral SS should be considered in the context that these patients also require fewer additional surgeries under general anaesthesia, thereby reducing overall demands on staffing and resource allocation. Notably, in the present study, complications to the symmetrized breast in the simultaneous SS group were all minor and treated conservatively, an important consideration, particularly in the setting of adjuvant oncological therapy. Finally, in the present study, 18 of 36 patients (51.4%) in the simultaneous contralateral SS group required additional surgery under general anaesthesia to complete breast reconstruction, reflecting the difficulties in achieving symmetry at the index surgery. These findings are consistent with those reported by Peltristo *et al*.^14^.

In the present study, women undergoing simultaneous contralateral SS were older than women undergoing secondary contralateral SS (mean(s.d.) age 55.5(8.6) *versus* 51.9(7.7) years; *P* = 0.036). It is unclear what accounts for this finding, although it is possible that older women had more ptotic breasts, making the indication for SS more apparent^25^.

The literature on PROs following contralateral SS in unilateral autologous breast reconstruction is limited, with few large prospective comparative studies. Existing studies are heterogeneous in terms of study populations and outcome assessments, but their findings are generally concordant with those of the present study. For example, Huang *et al*.^26^ used a modified questionnaire from Heden *et al*.^27^ in their study examining patient satisfaction of simultaneous contralateral balancing mastopexy/reduction patients with unilateral abdominal-based free flap breast reconstruction. In that study^26^, 16 of 22 participants completed the survey, and all participants described the whole breast reconstructive procedure with simultaneous symmetrization as ‘very advantageous’ or ‘quite advantageous’. Questions pertaining to physical health were generally scored as ‘much better’ or ‘quite better’, whereas half the patients answered questions pertaining to interpersonal relationships and intimacy as ‘unchanged’. In another study, Inbal *et al*.^15^ reported findings for 51 patients undergoing unilateral deep inferior epigastric perforator breast reconstruction, eight of whom underwent secondary SS, 33 of whom underwent simultaneous SS, and 10 of whom underwent no SS (control group). The BREAST-Q™ questionnaire was administered at an unspecified time after breast reconstruction. Inbal *et al*.^15^ found no difference in either satisfaction (breast, abdomen, outcome) or wellbeing (sexual, physical, psychosocial, abdomen) scales between the secondary and simultaneous SS groups. However, they did report that both psychosocial wellbeing and quality of life scores were reduced in the secondary SS group during the latent phase between index breast reconstruction and SS^15^, in line with the findings of the present study. Finally, Bitoiu *et al*.^28^ compared preoperative and postoperative BREAST-Q™ scores in women with unilateral breast cancer undergoing mastectomy and contralateral symmetrization either with classical techniques (reduction, mastopexy, augmentation) or with mastectomy and reconstruction, in essence bilateral breast reconstruction. Both autologous free flap and implant-based reconstructive methods were included. With 3- and 12-month postoperative response rates of 18.9% (47 patients) and 15.3% (38), respectively, Bitoiu *et al*.^28^ found lower scores for physical wellbeing chest wall in patients with SS preformed using classical techniques compared with contralateral mastectomy with reconstruction.

The present study adds valuable insights by using the validated BREAST-Q™ questionnaire at multiple timepoints, with high response rates at baseline. A strength of the study design is the use of both early (3 months) and long-term (2 years) PROs to examine trends over time. However, several limitations should be considered. First, the postoperative response rates were modest (overall 64.8% at 3 months and 52.8% at 2 years), which may introduce response bias. Second, the observational study design limits the ability to establish causality. Third, early improvements in satisfaction with breasts in the simultaneous contralateral SS group may partly reflect overall satisfaction with the index operation rather than the specific effect of the contralateral procedure. In addition, the relatively small sample size may have reduced the power to detect differences in complication rates or conduct robust subgroup analyses. The inclusion of both immediate and delayed breast reconstructions could also introduce bias; to address this, the immediate cohort was excluded from comparative analyses of PRO measures. Finally, the single-centre study design may limit the generalizability of the findings.

Future studies with larger sample sizes are needed to assess whether specific patient groups (for example, those receiving adjuvant chemotherapy) may benefit more from one approach over another. Furthermore, resource allocation and logistical aspects of performing simultaneous *versus* secondary SS, such as operating time, hospital length of stay, and cost, warrant further investigation.

## Supplementary Material

zrag023_Supplementary_Data

## Data Availability

The data are not publicly available due to [reasons] but are available from the corresponding author upon reasonable request.
