# Supplementary material for: Effect of timing on patient-reported outcomes in contralateral symmetrization surgery in autologous breast reconstruction
Source: BJS Open. 2026 May 13;10(3):zrag023. doi: 10.1093/bjsopen/zrag023 (PMC13167258; doi:10.1093/bjsopen/zrag023)

**Contralateral symmetrisation surgery in autologous breast reconstruction: does timing affect patient reported outcomes?**

Rojda Gümüscü MD^1, 2*^, Olivia Sjökvist MD^1,2 *#^, Ellen Kragsterman MD^2^, Johan Svensson PhD^3^, Susanna Kauhanen MD PhD^1,4^, Maria Mani MD PhD^1, 2^, Rebecca Wiberg MD, PhD^5^

^1^Department of Surgical Sciences, Faculty of Medicine, Uppsala University, Uppsala, Sweden

^2^ Department of Plastic and Maxillofacial Surgery, Uppsala University Hospital, Uppsala, Sweden

^3^Department of Statistics, Umeå School of Business, Economics and Statistics, Umeå University, Umeå, Sweden.

^4^ Department of Plastic Surgery, University of Helsinki and Helsinki University Hospital, Helsinki, Finland

^5^ Department of Diagnostics and Intervention, Plastic Surgery and Surgery, Umeå University, Umeå, Sweden.

* Shared co-first authorship. The second author have contributed equally to the publication

**^#^ Corresponding author:**

**Olivia Sjökvist**

Address: Department of Plastic and Maxillofacial Surgery, Sjukhusvägen, 751 85 Uppsala, Sweden

Email: olivia.sjokvist@uu.se

ORCID: 0000-0002-0172-9173

**Supplementary Materials - Index**

| **Supplementary Tables and Figures** |  |
| --- | --- |
| Supplementary Table 1. | *Page 3* |
| **Supplementary Methods** |  |
| BREAST-Q Reconstructive Module (version 1.0) | *Page 4* |
| BREAST-Q Scoring Sheet | *Page 13* |

**Supplementary Figures and Tables**

**Supplementary Table 1. BREAST-Q scores at baseline and at 3 months and two years post-operatively.**

**Supplementary Table 1.** *In the secondary cohort, all women were waiting for symmetrisation surgery at three months follow-up, ** while all women had undergone symmetrisation surgery two years post-operatively. Women that underwent immediate breast reconstruction were excluded from this analysis (n=7).

**Supplementary Methods**

BREAST-Q Reconstructive Module (version 1.0)

**
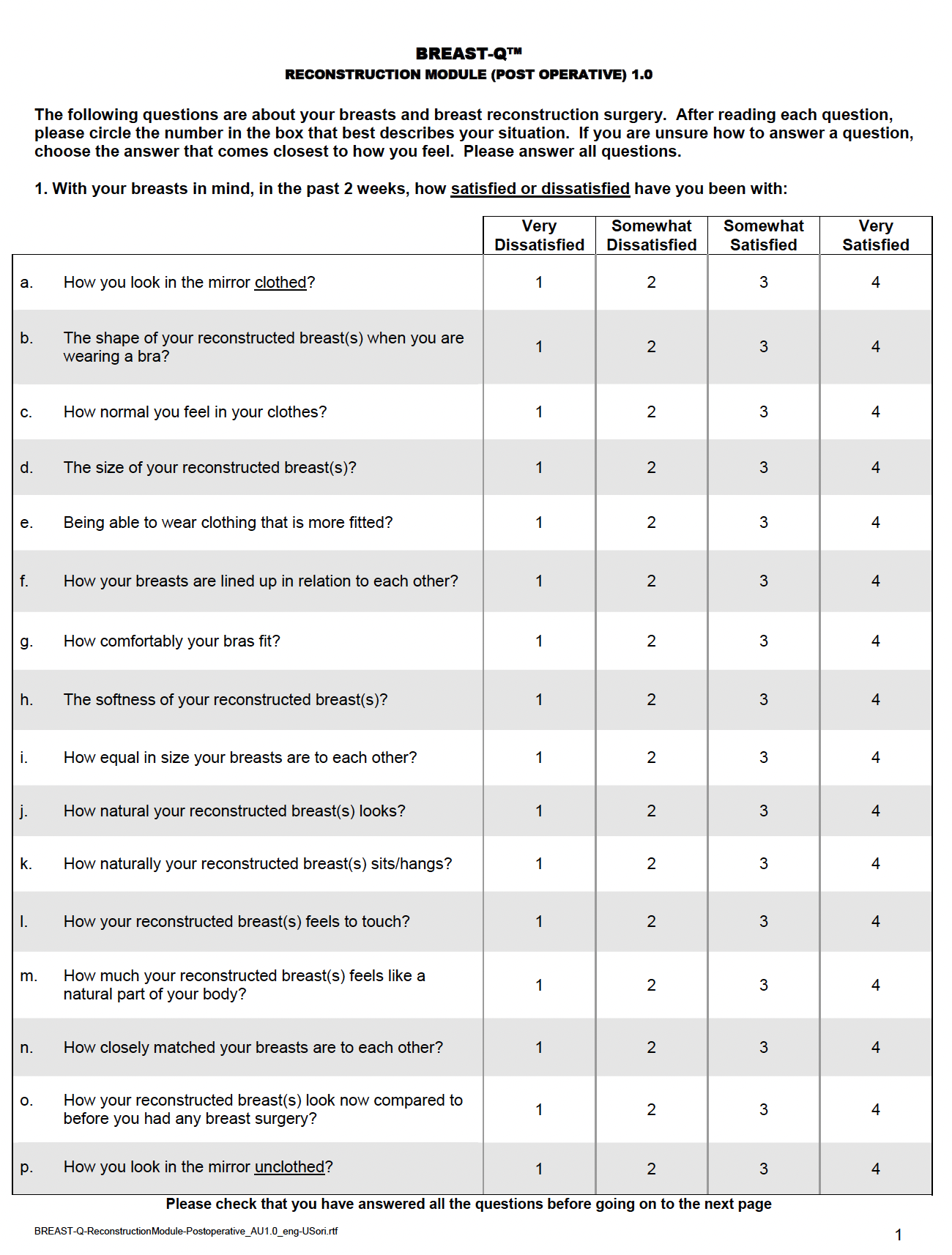
**

**
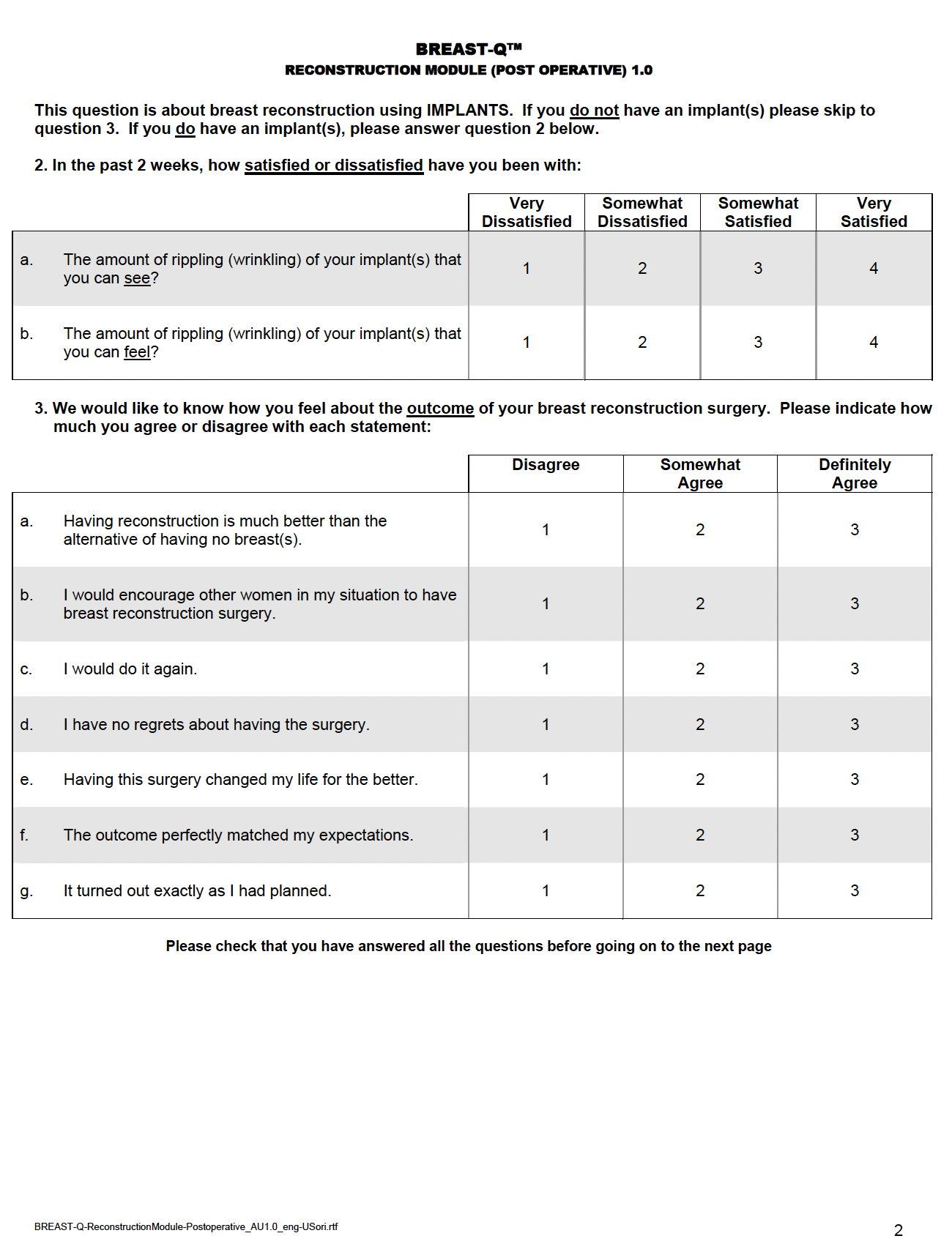
**

**
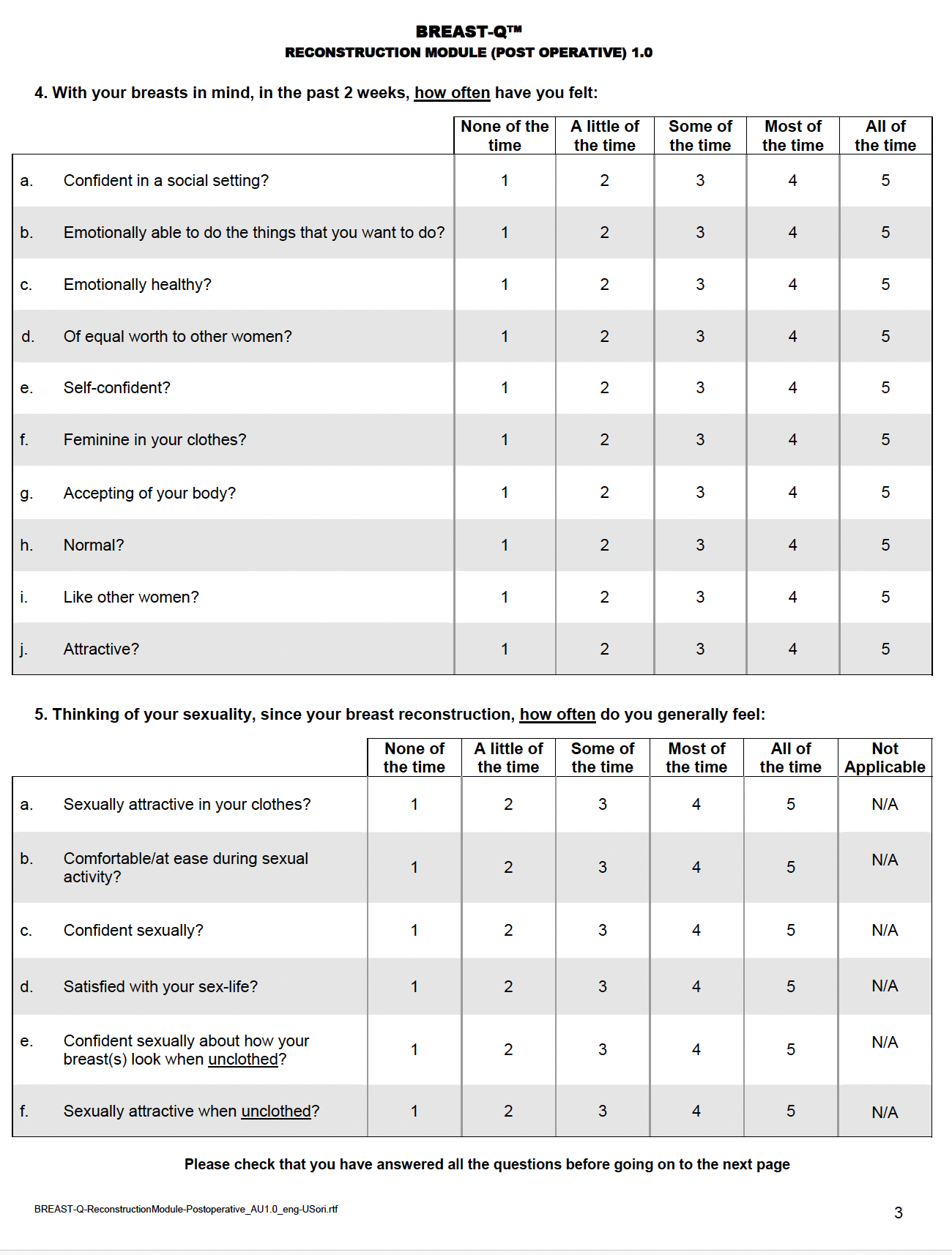
**

**
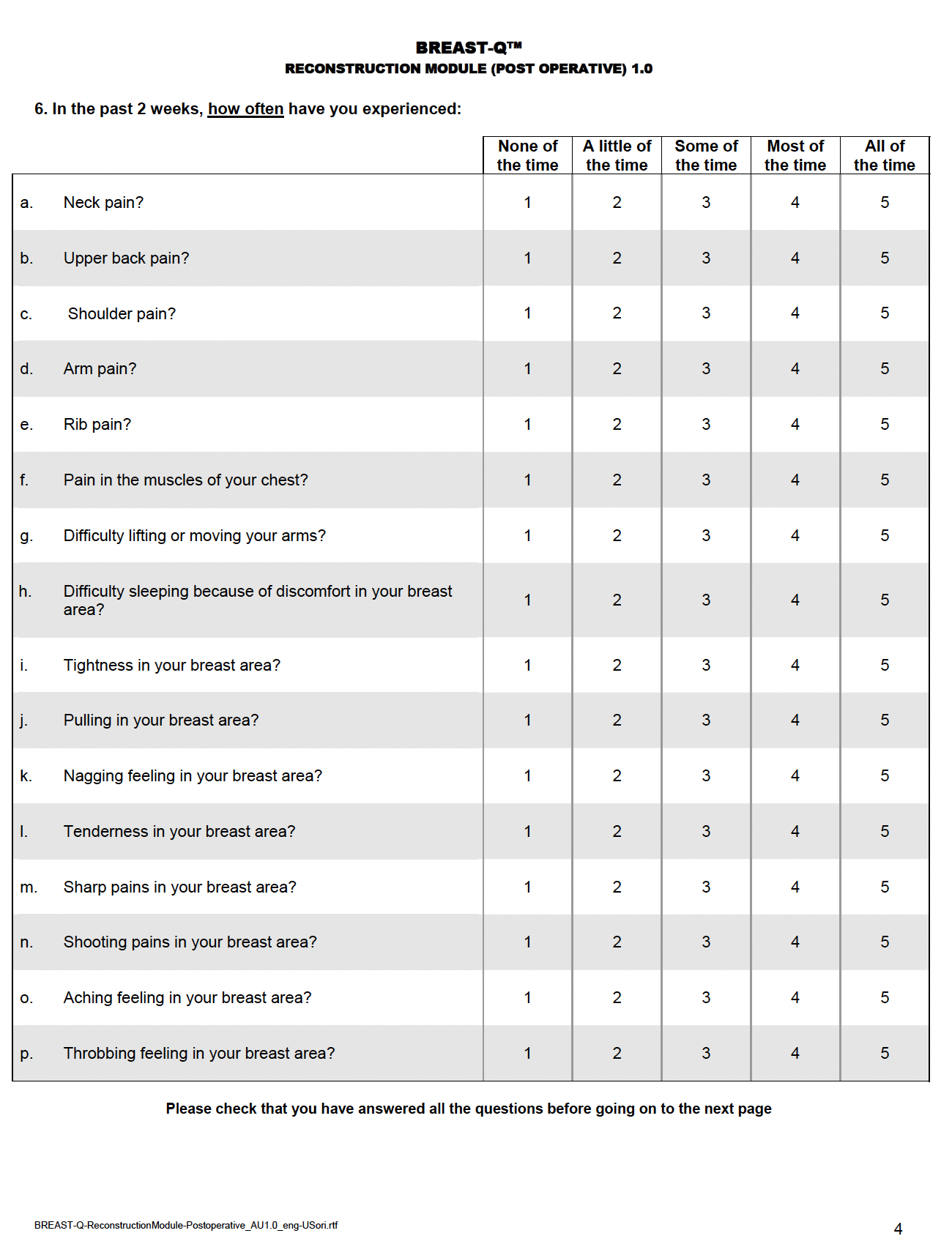
**

**
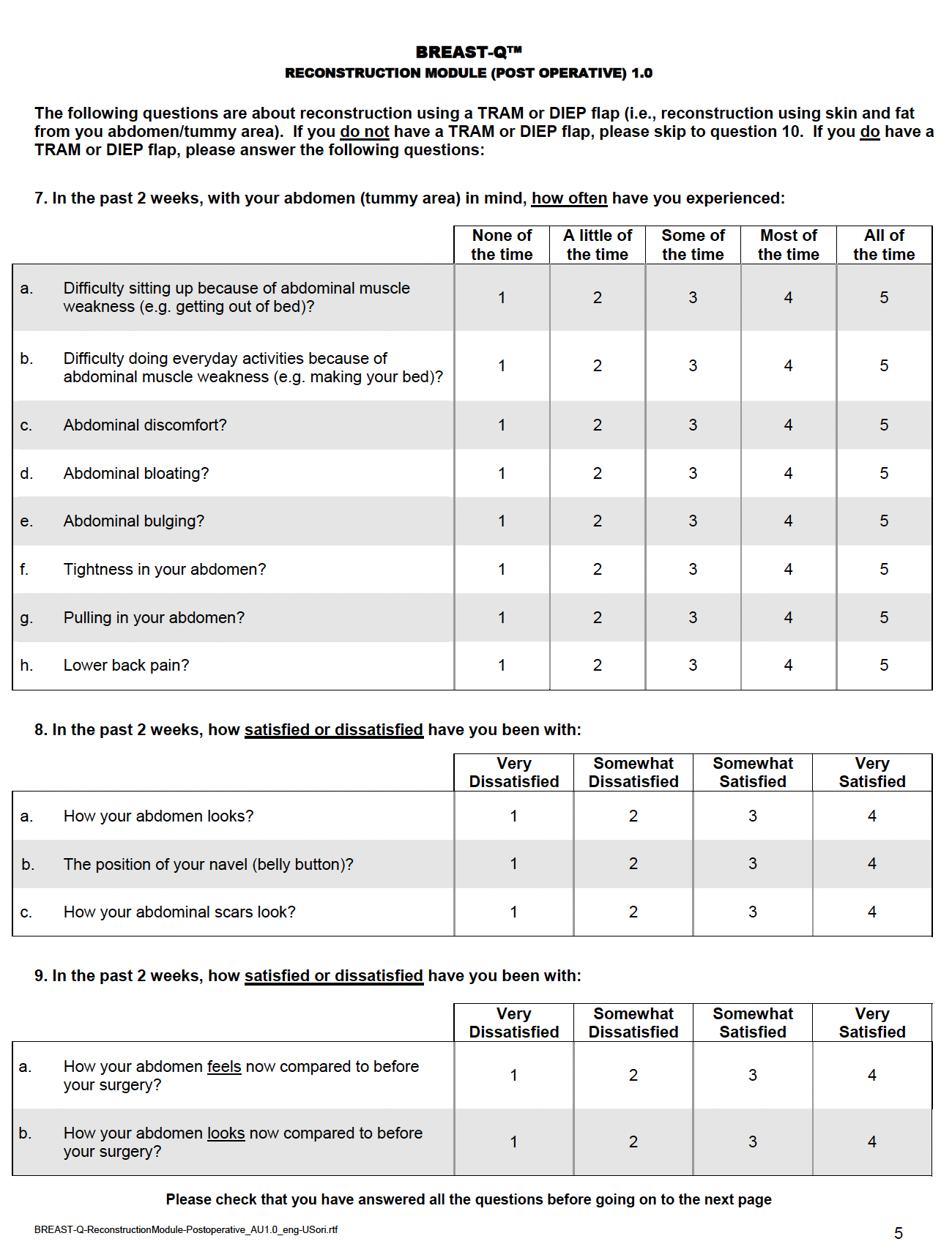
**

**
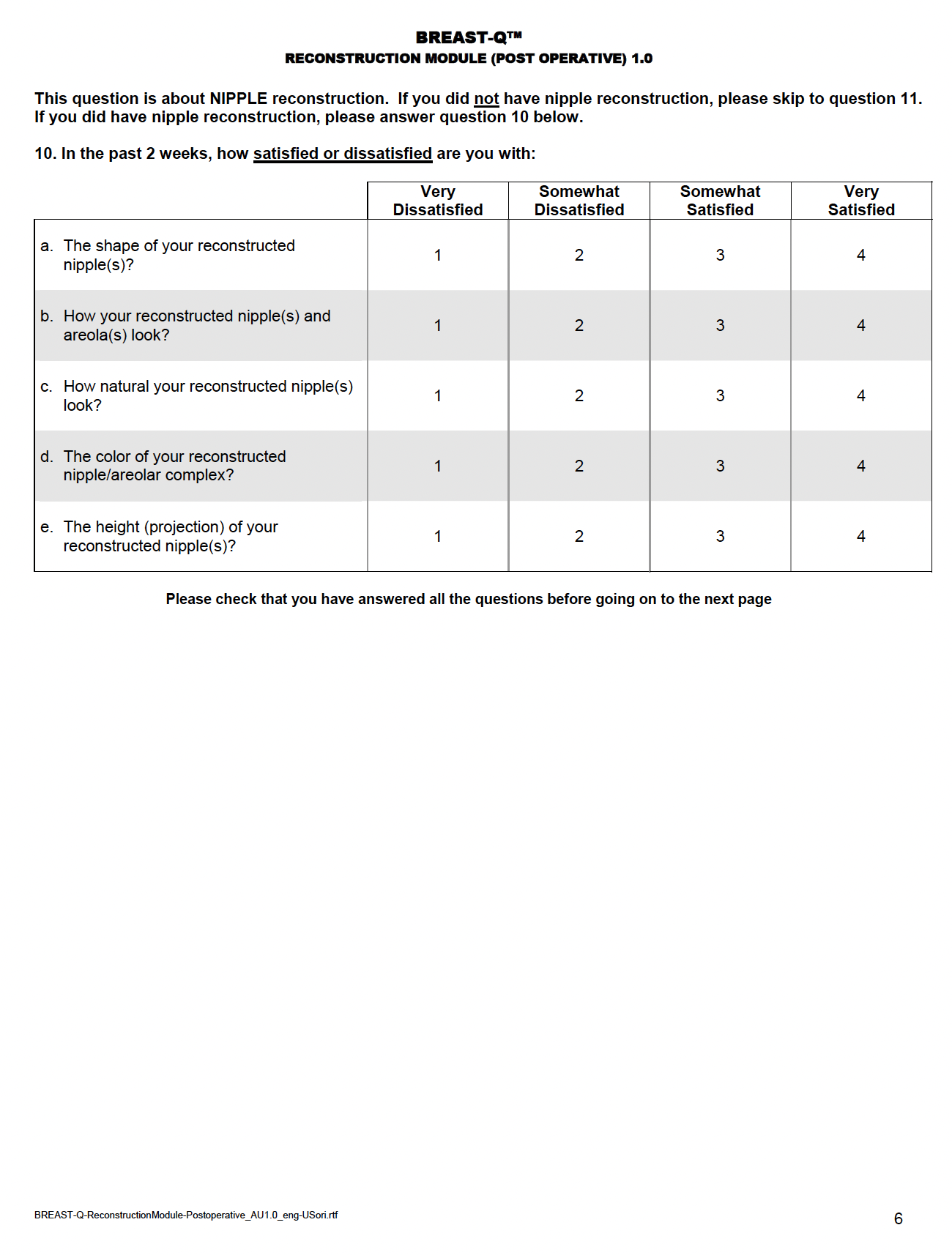
** **
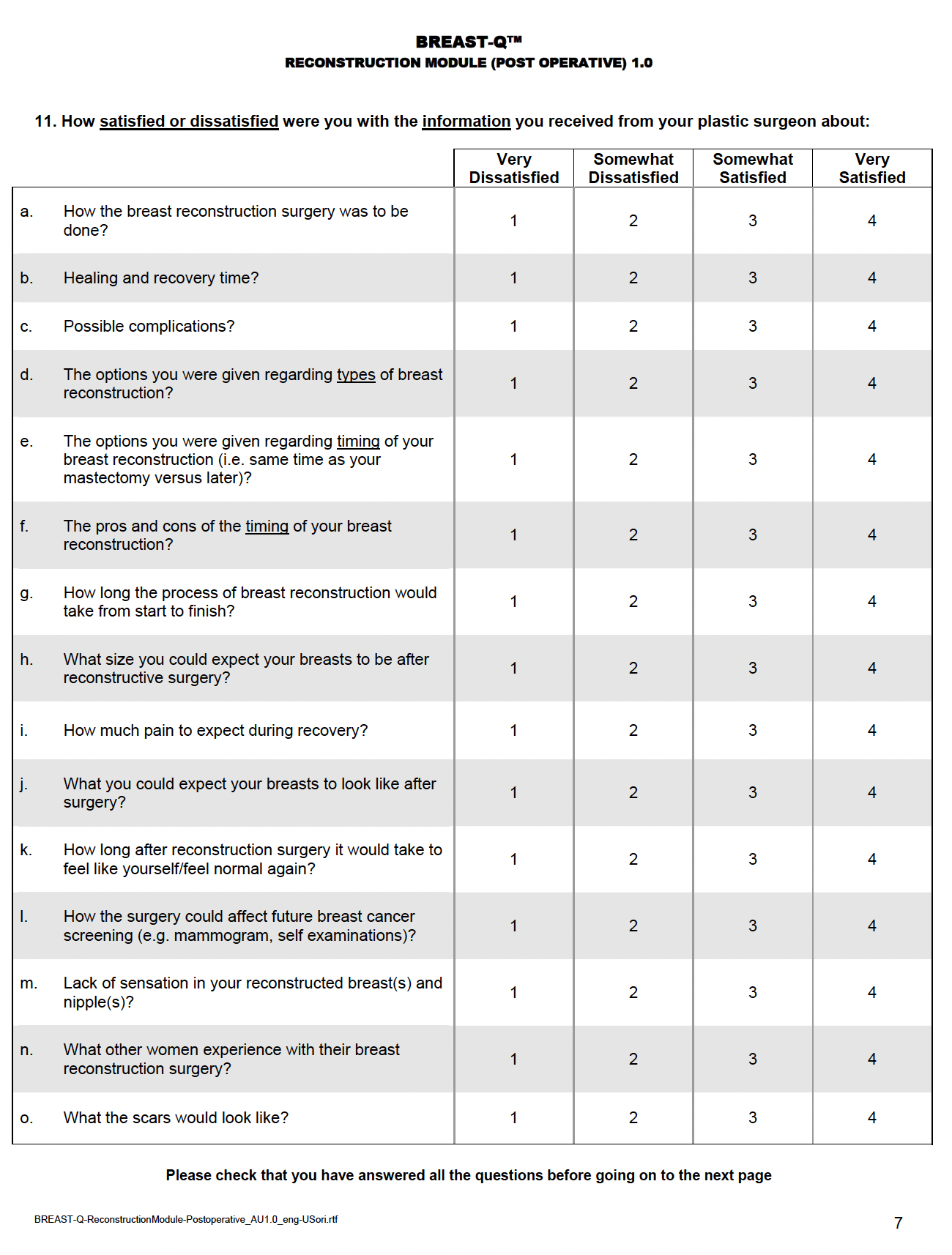
**
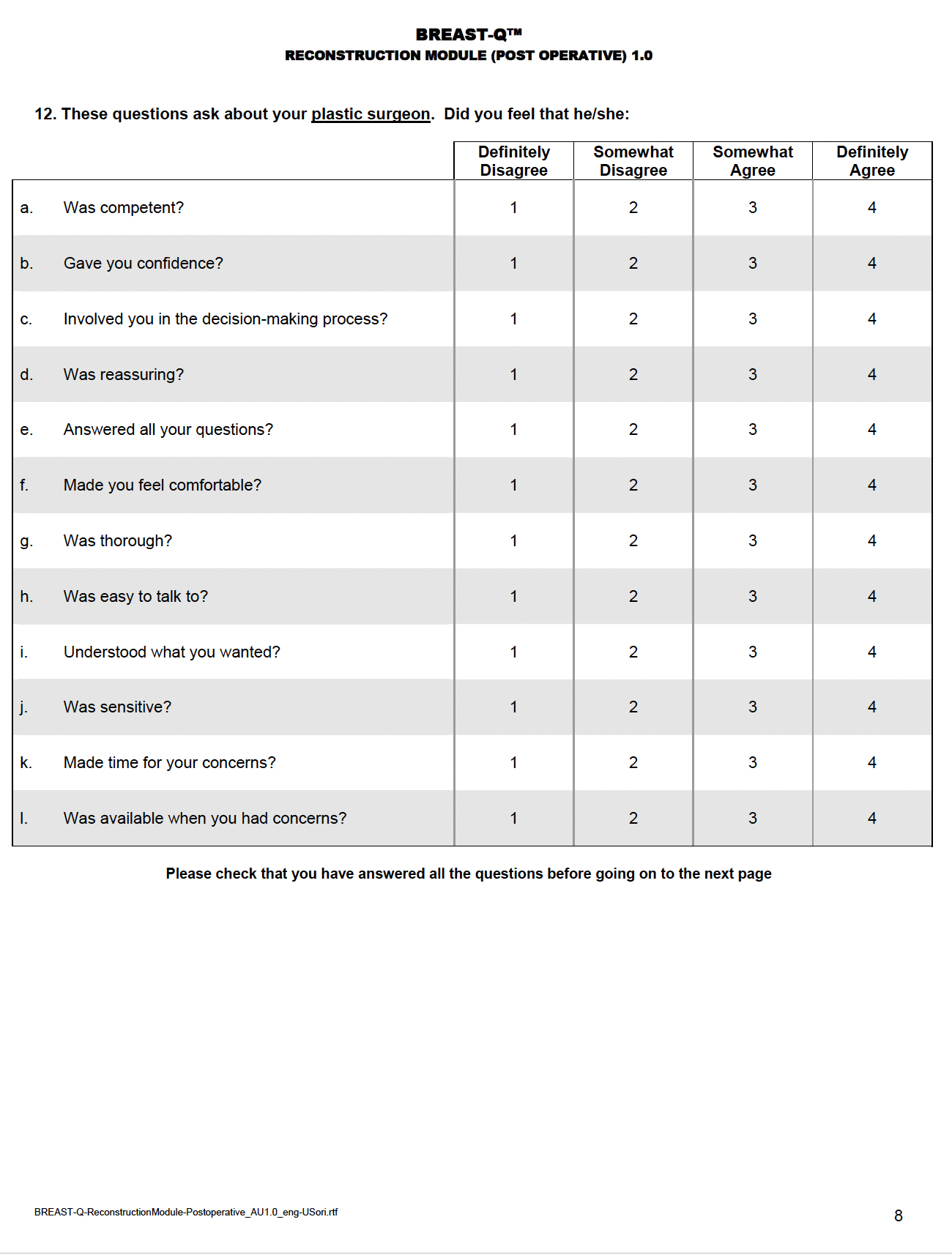


**
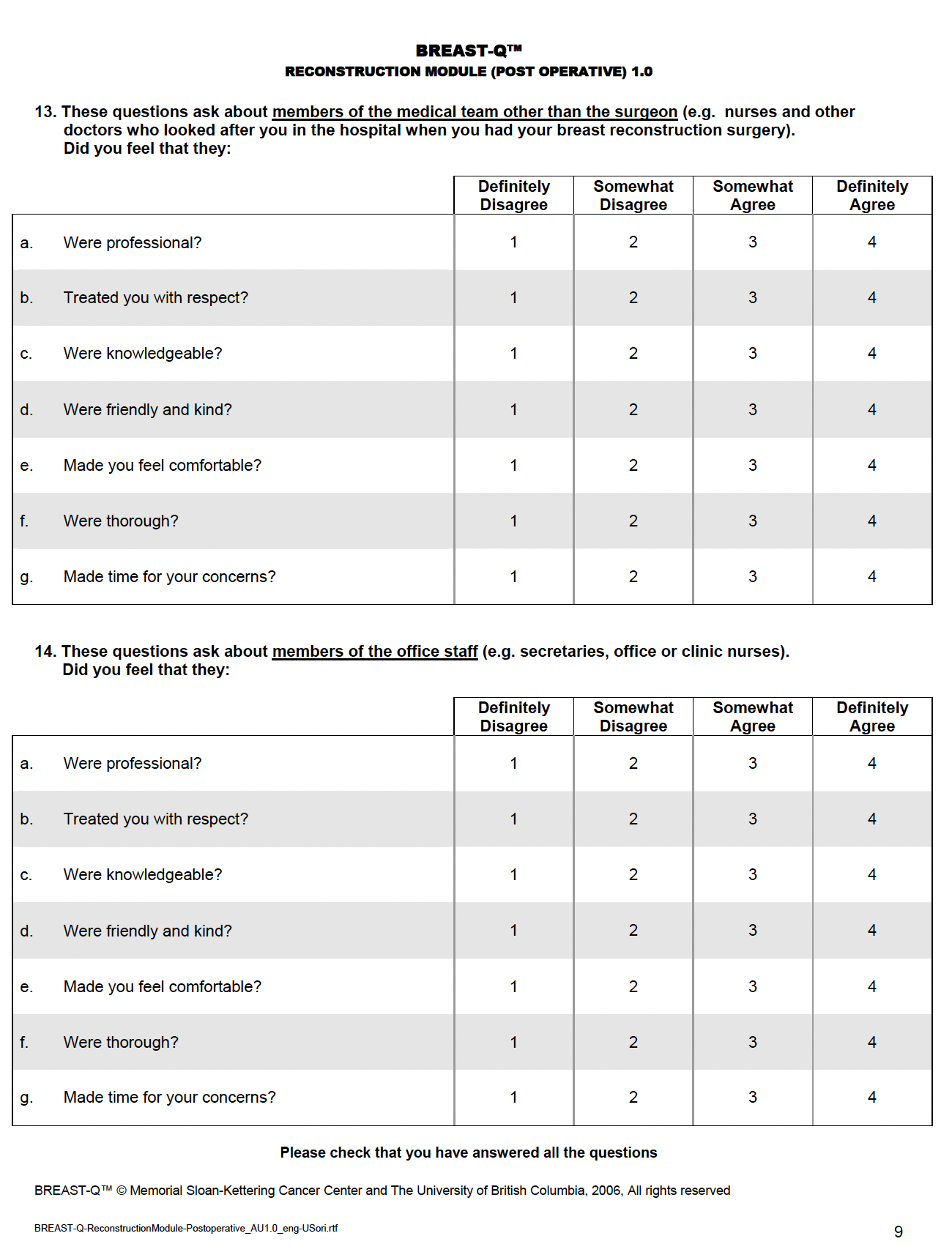
**

BREAST-Q Reconstructive Module Scoring Sheet (version 1.0)

**
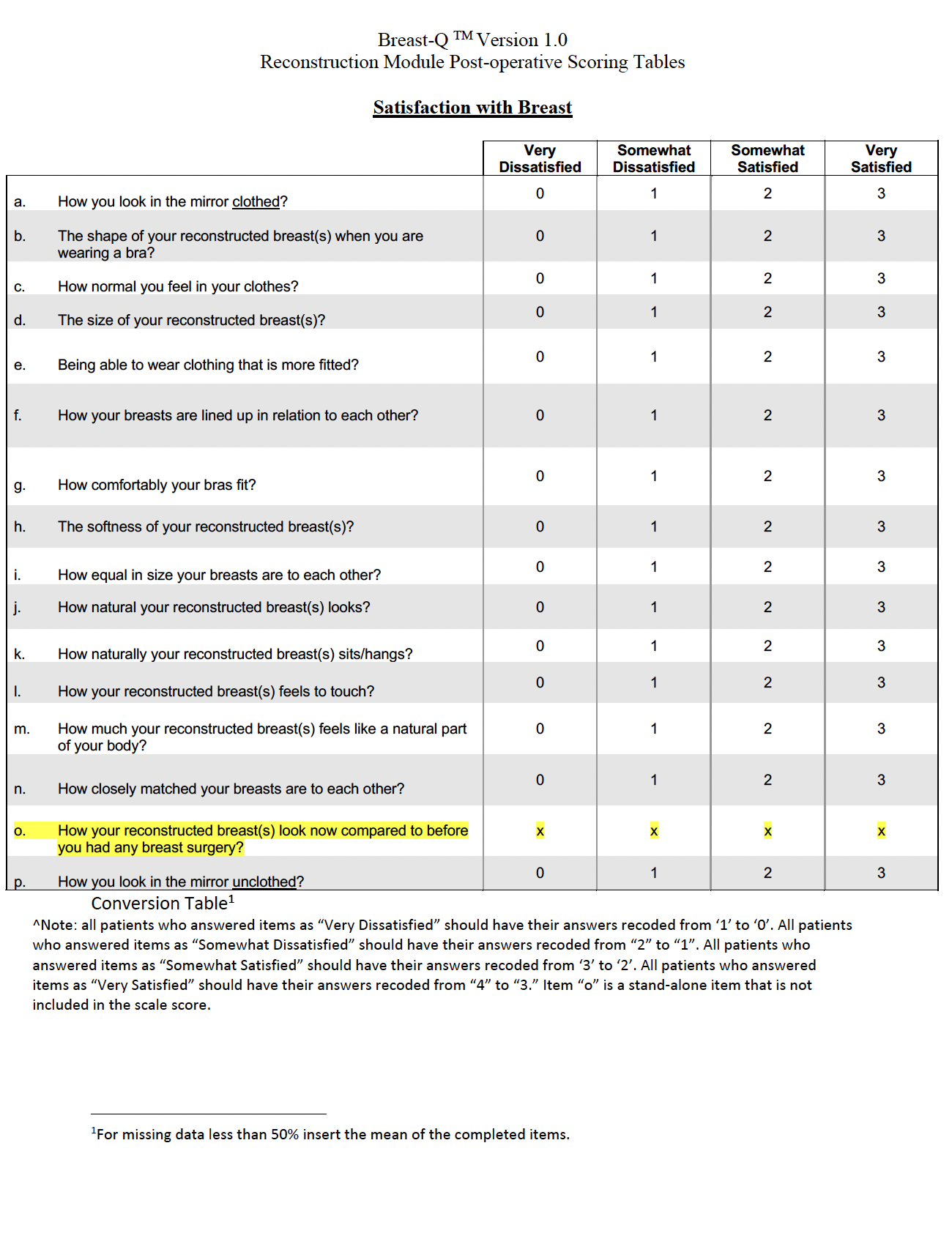
**

**
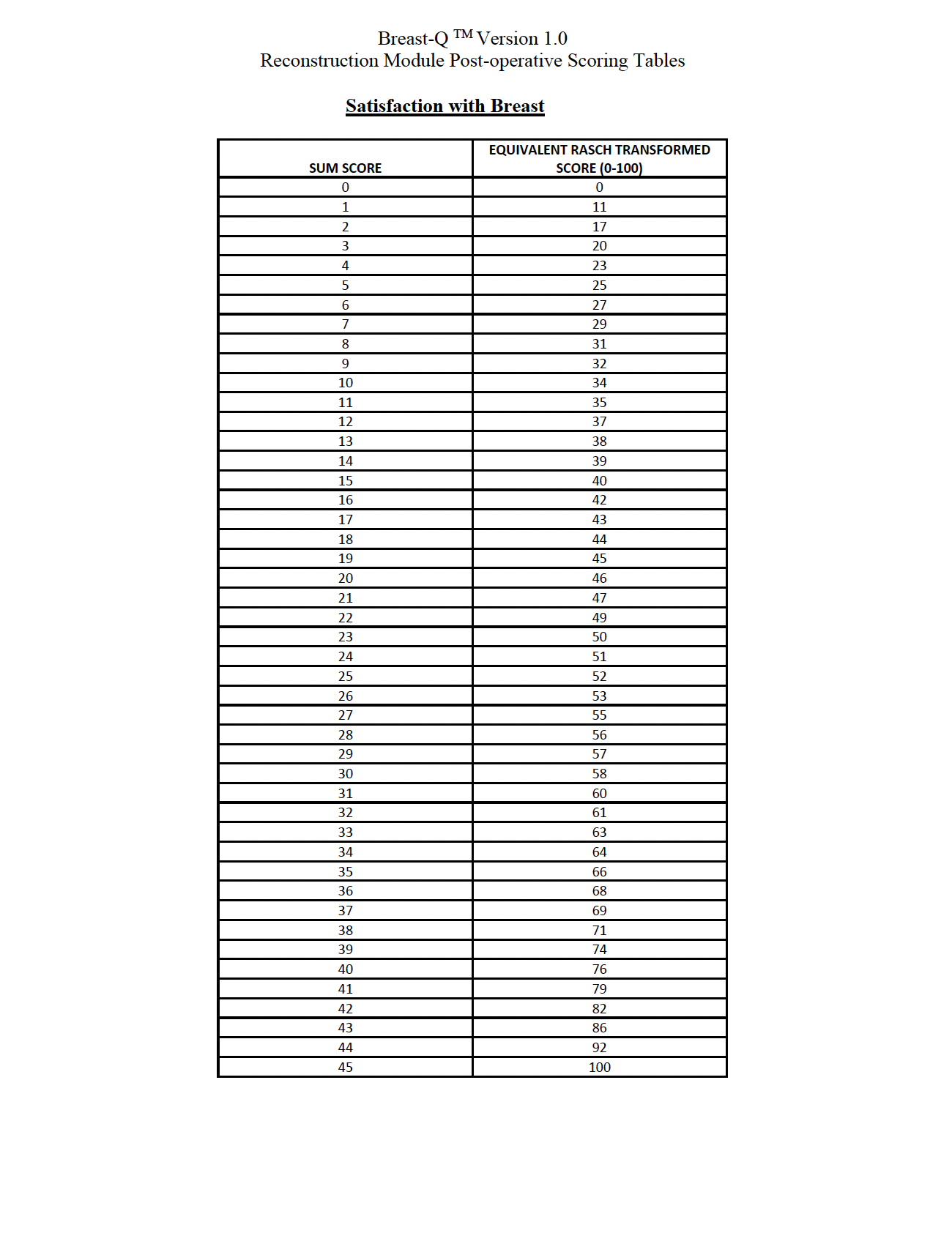
** **
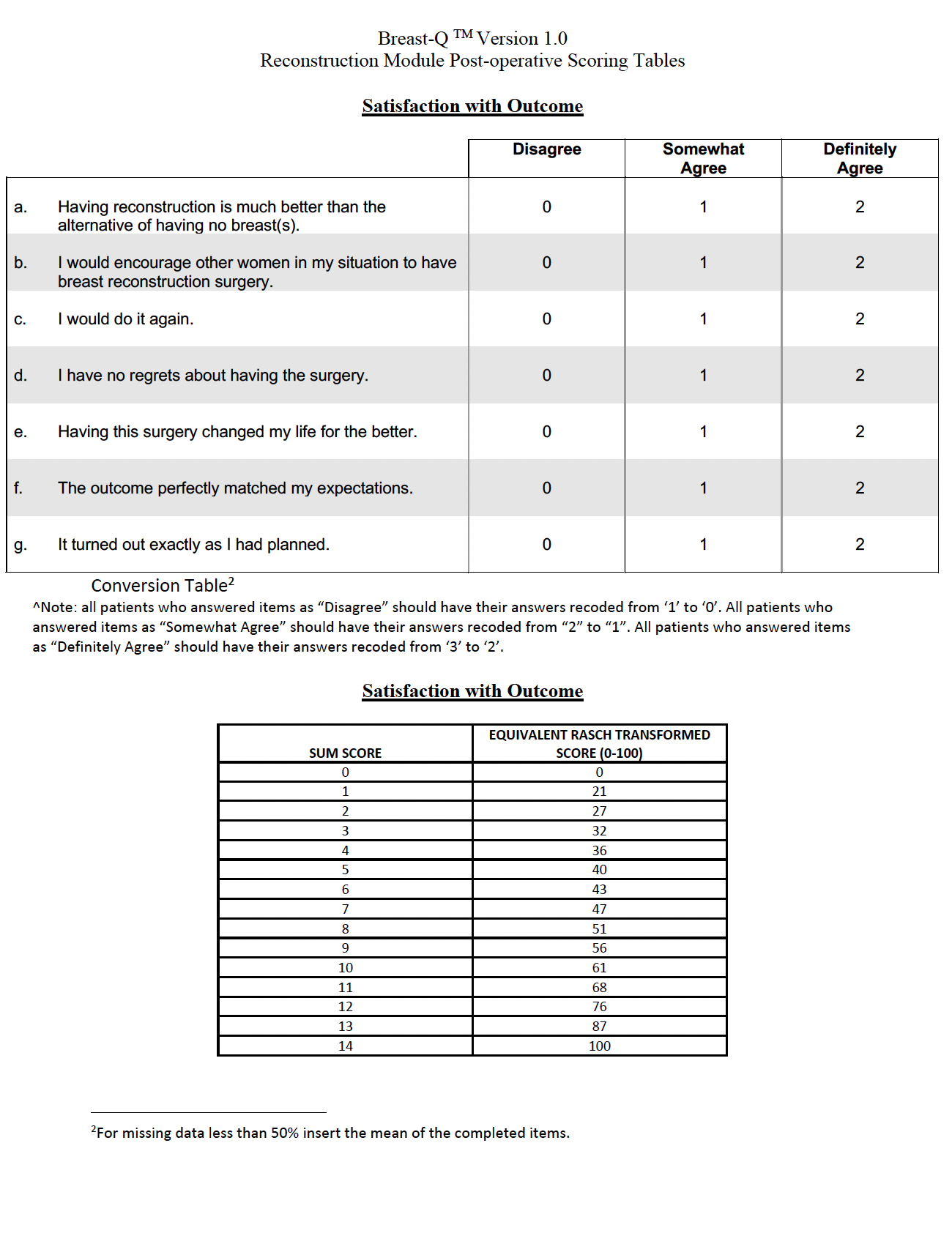
**
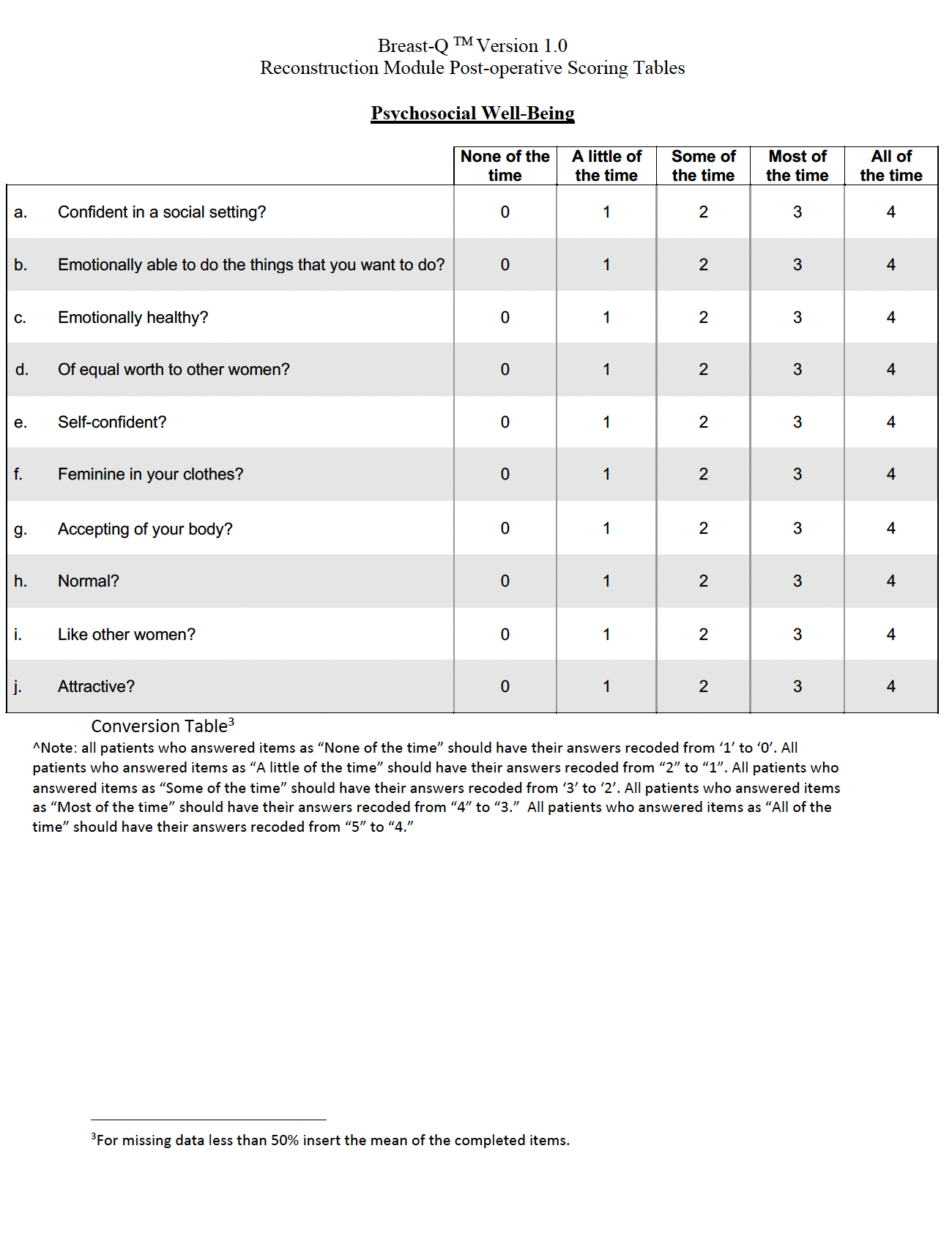

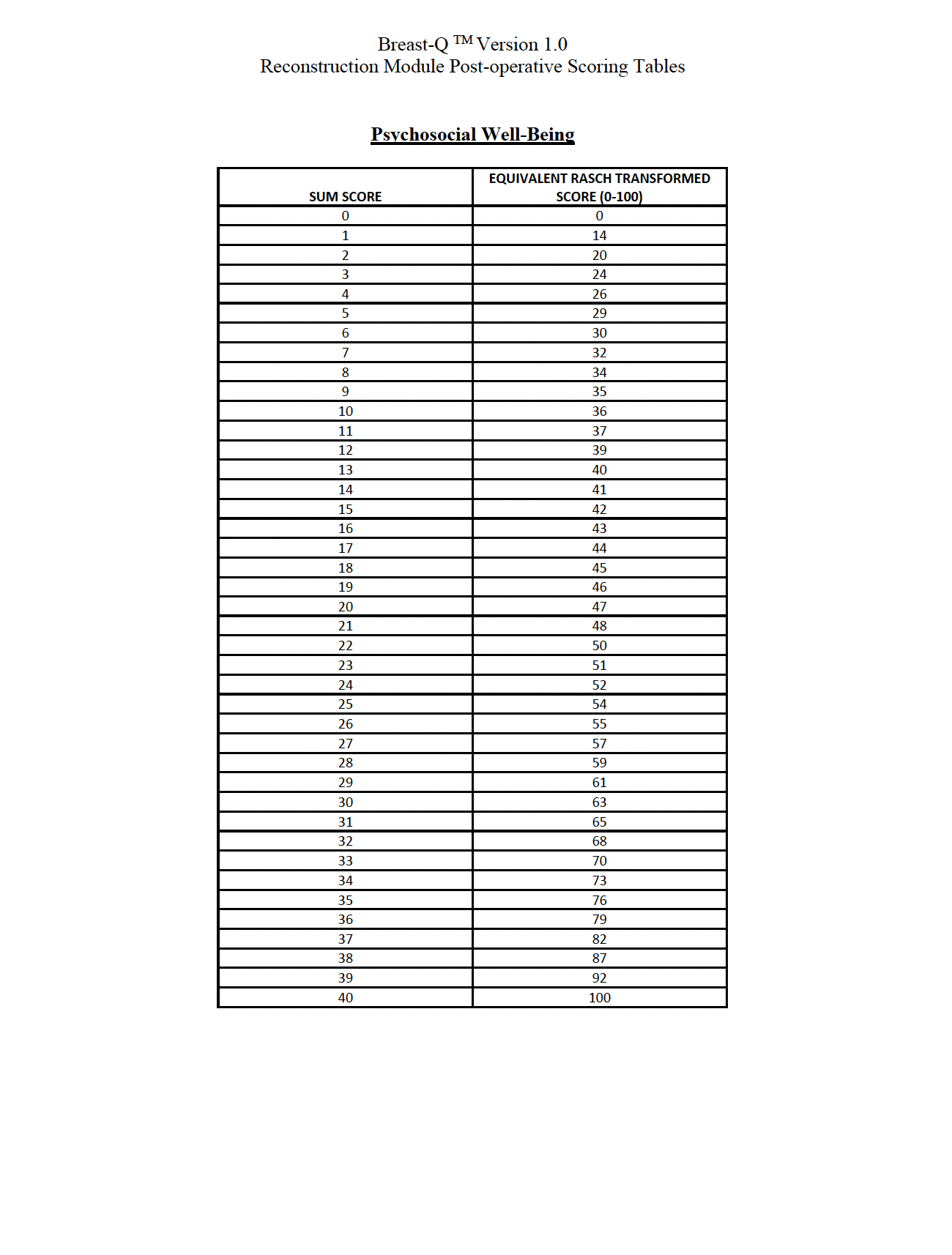

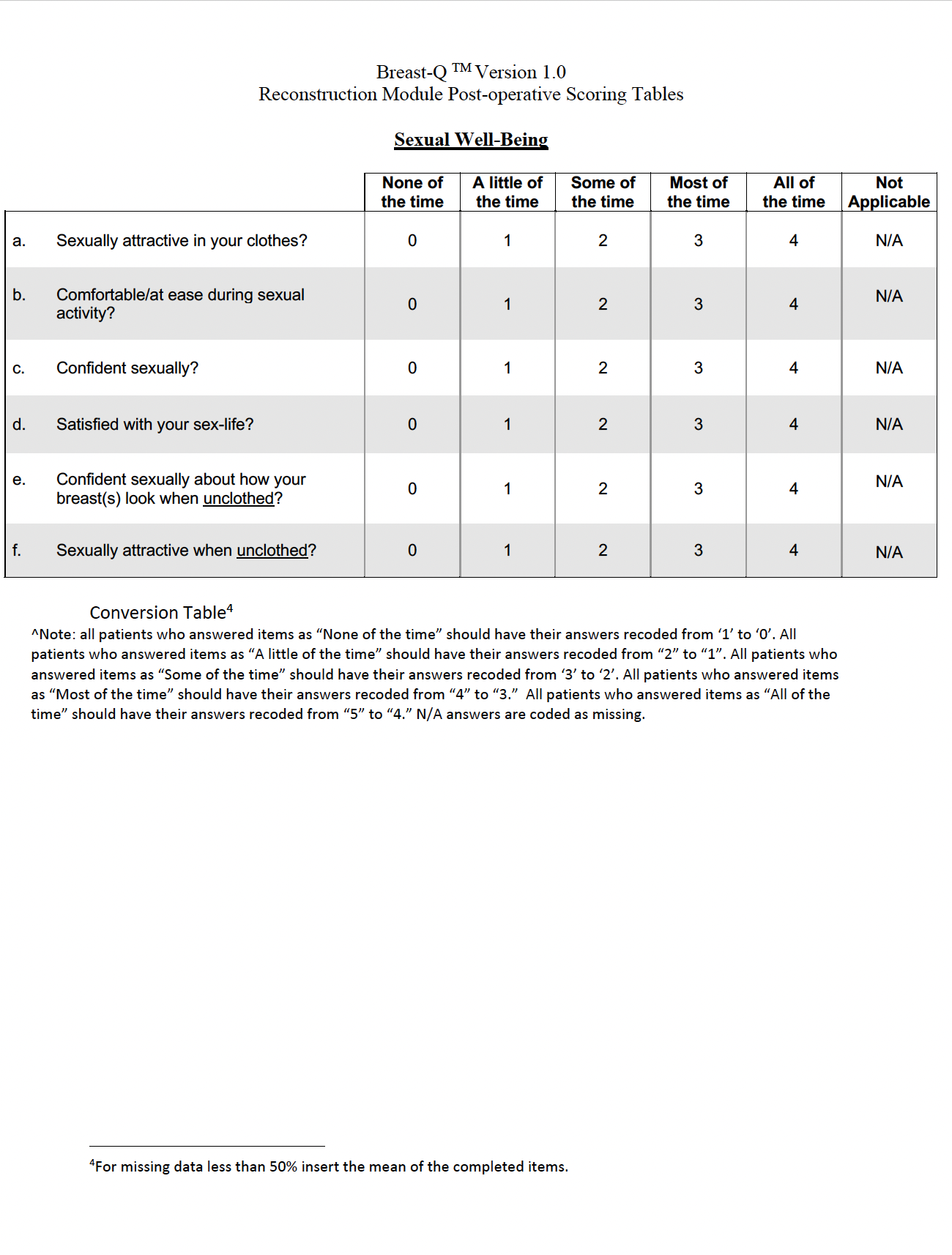

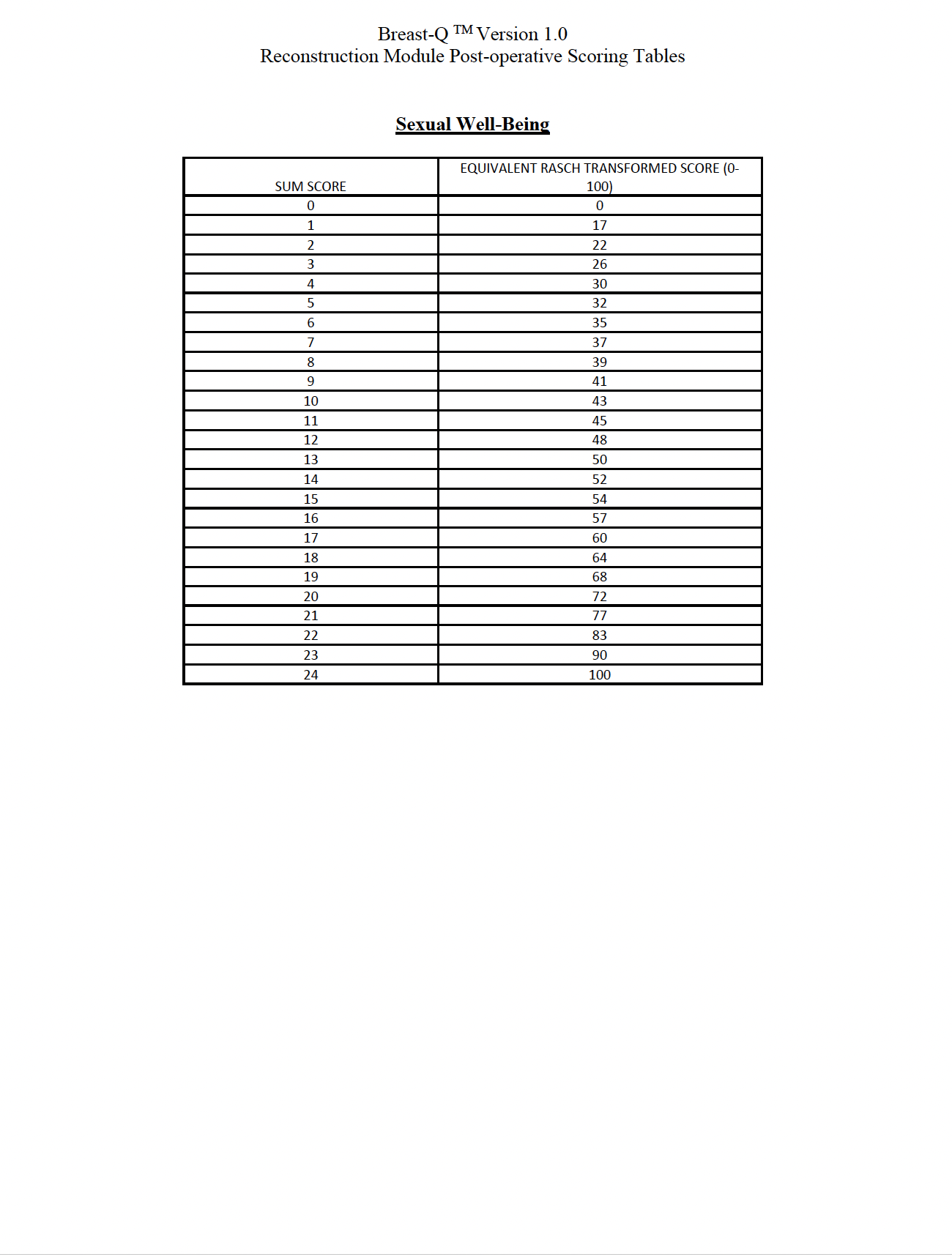

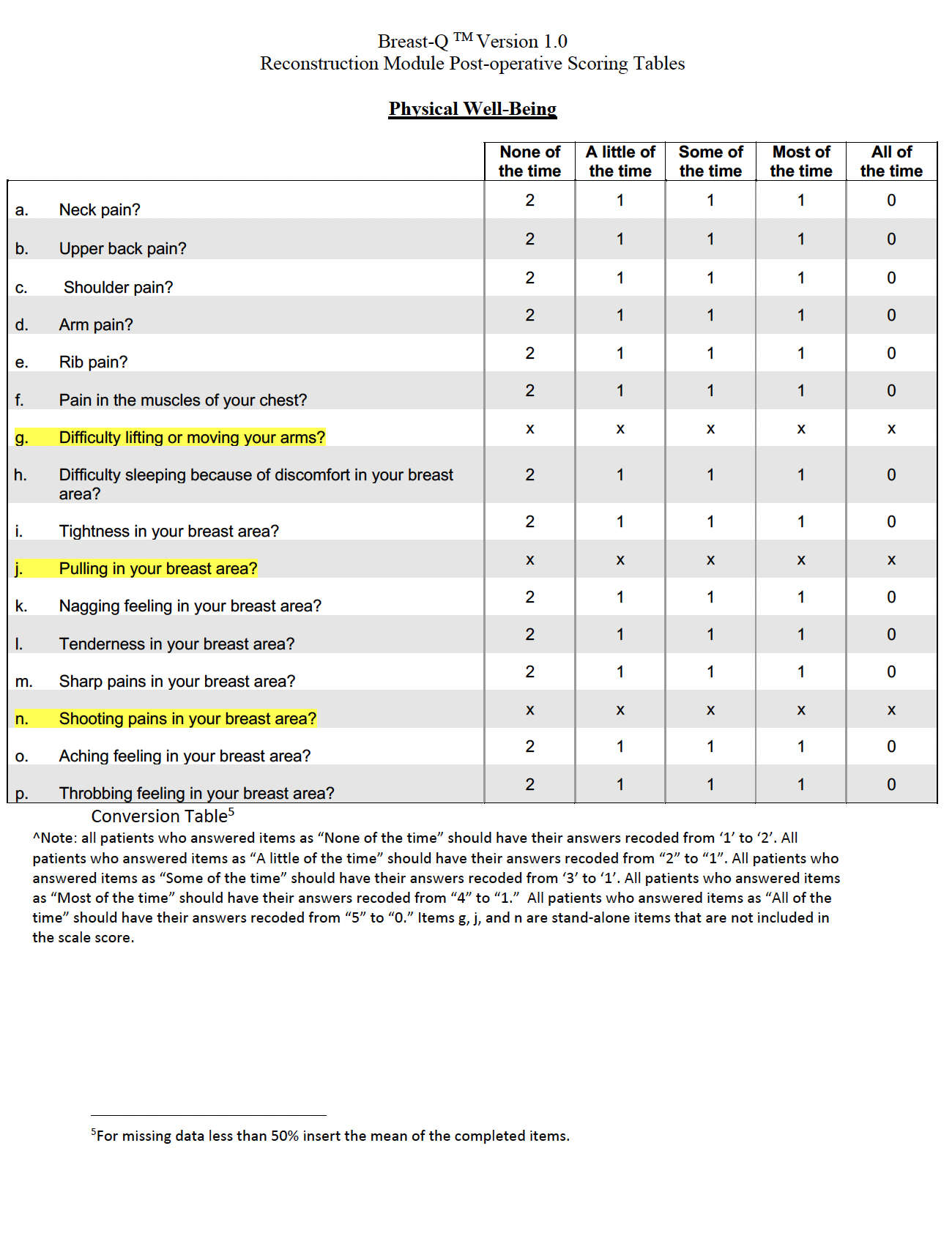

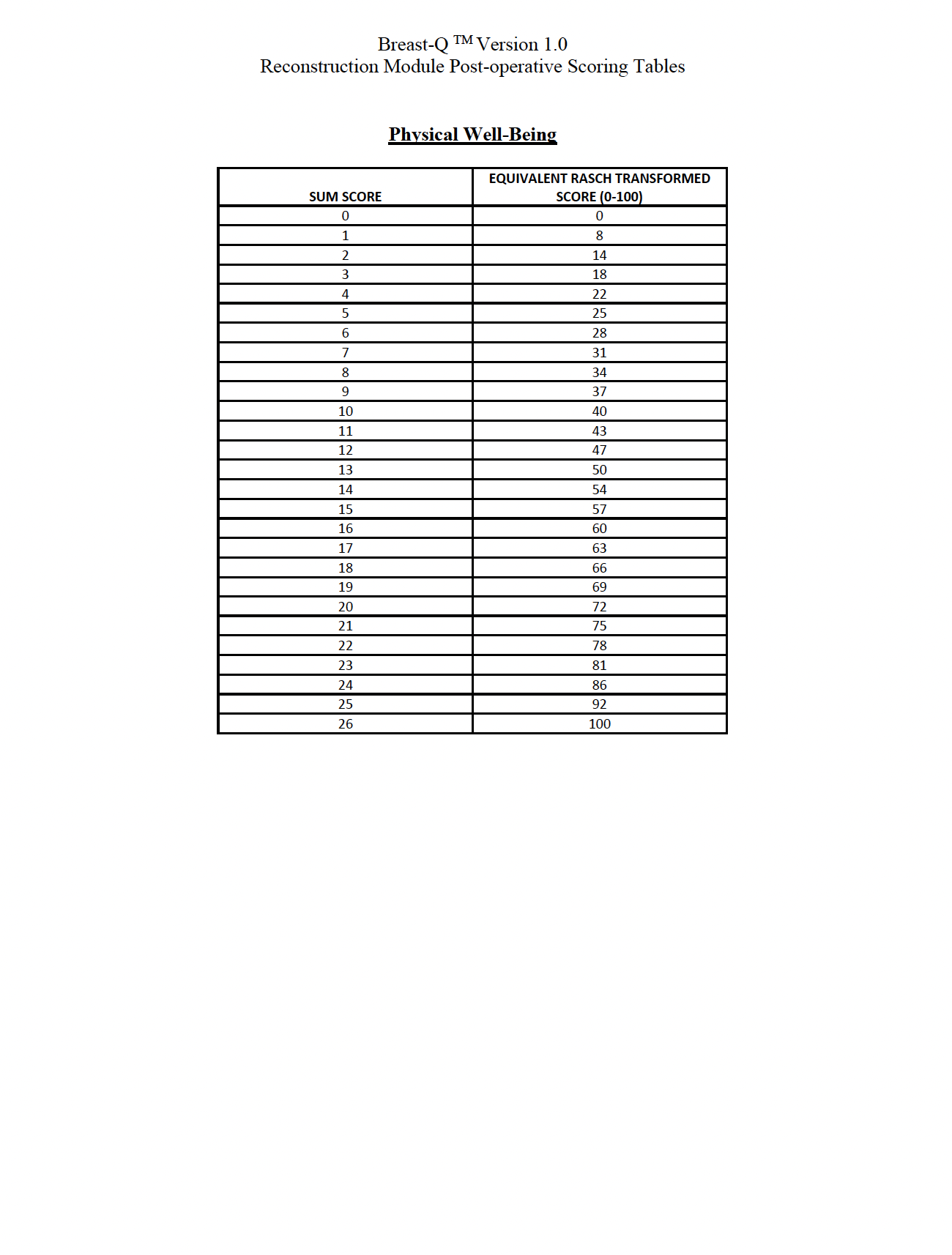

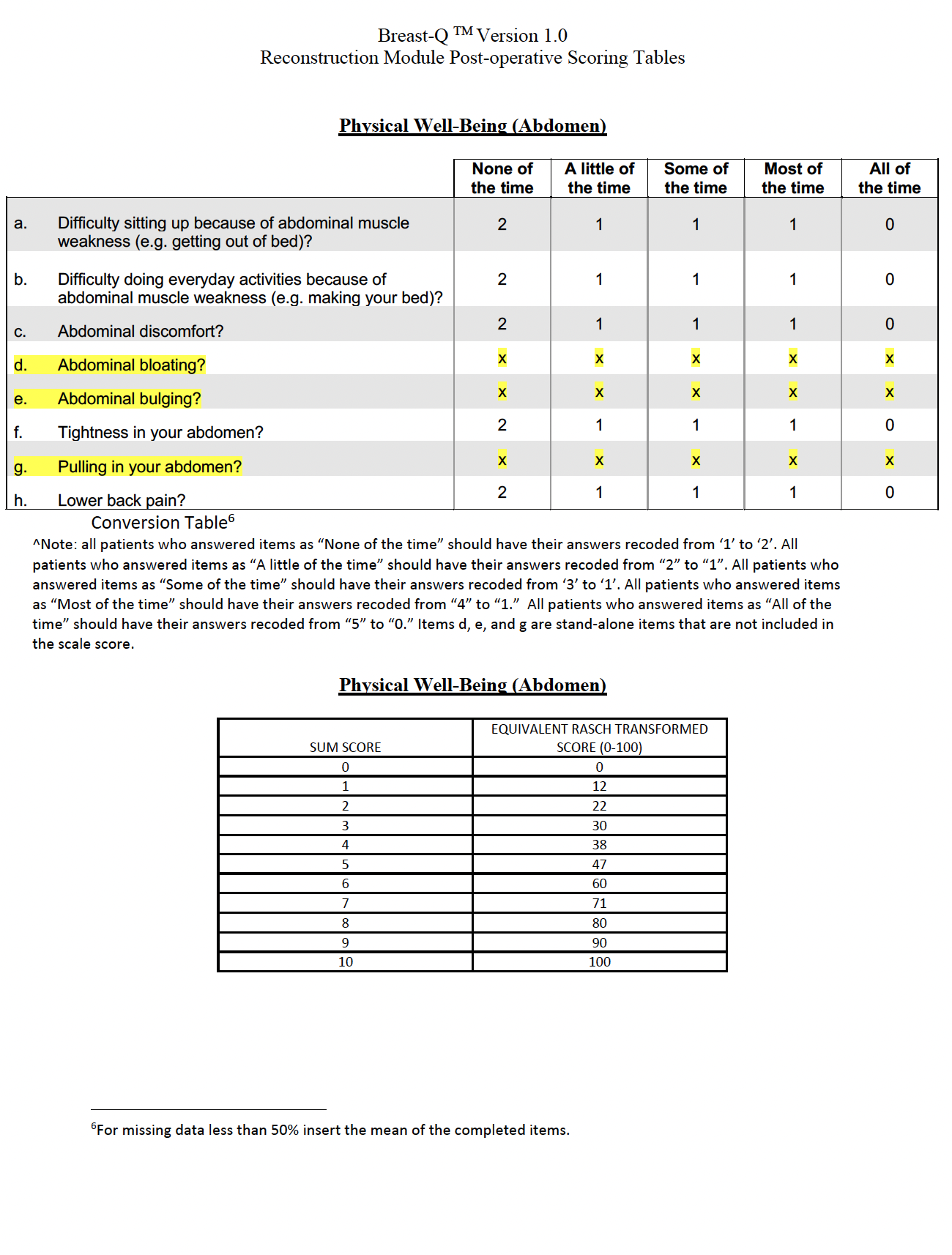

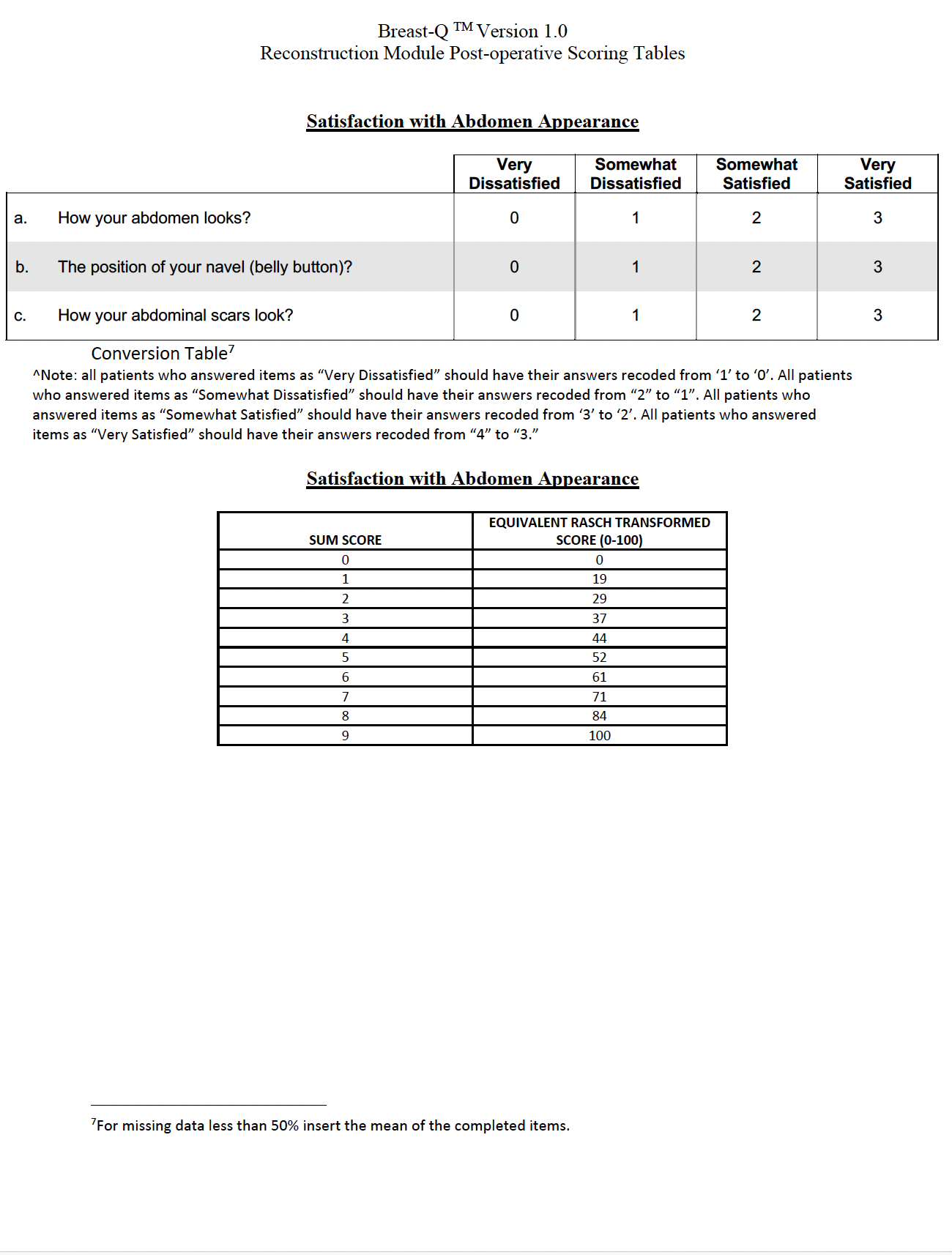

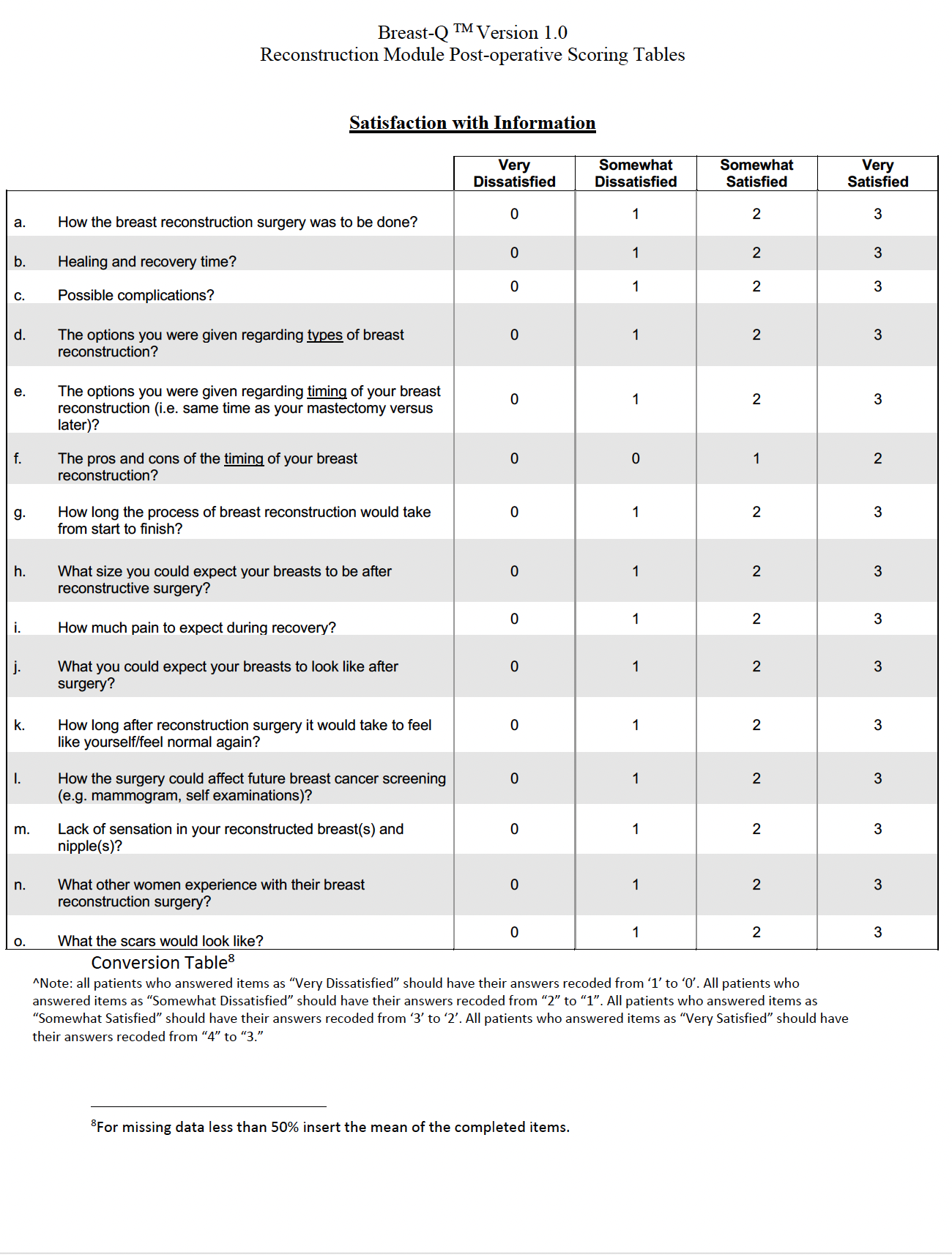

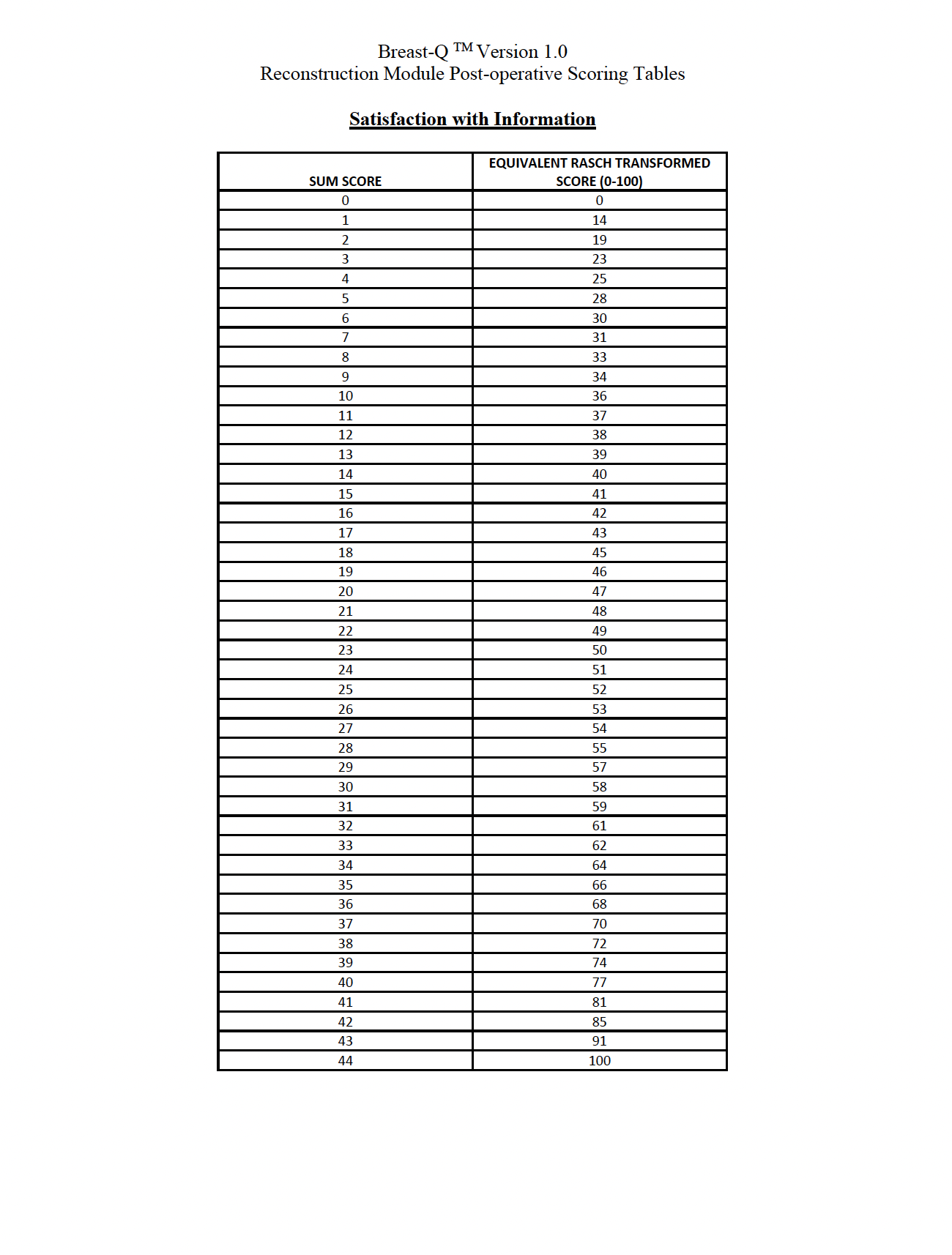

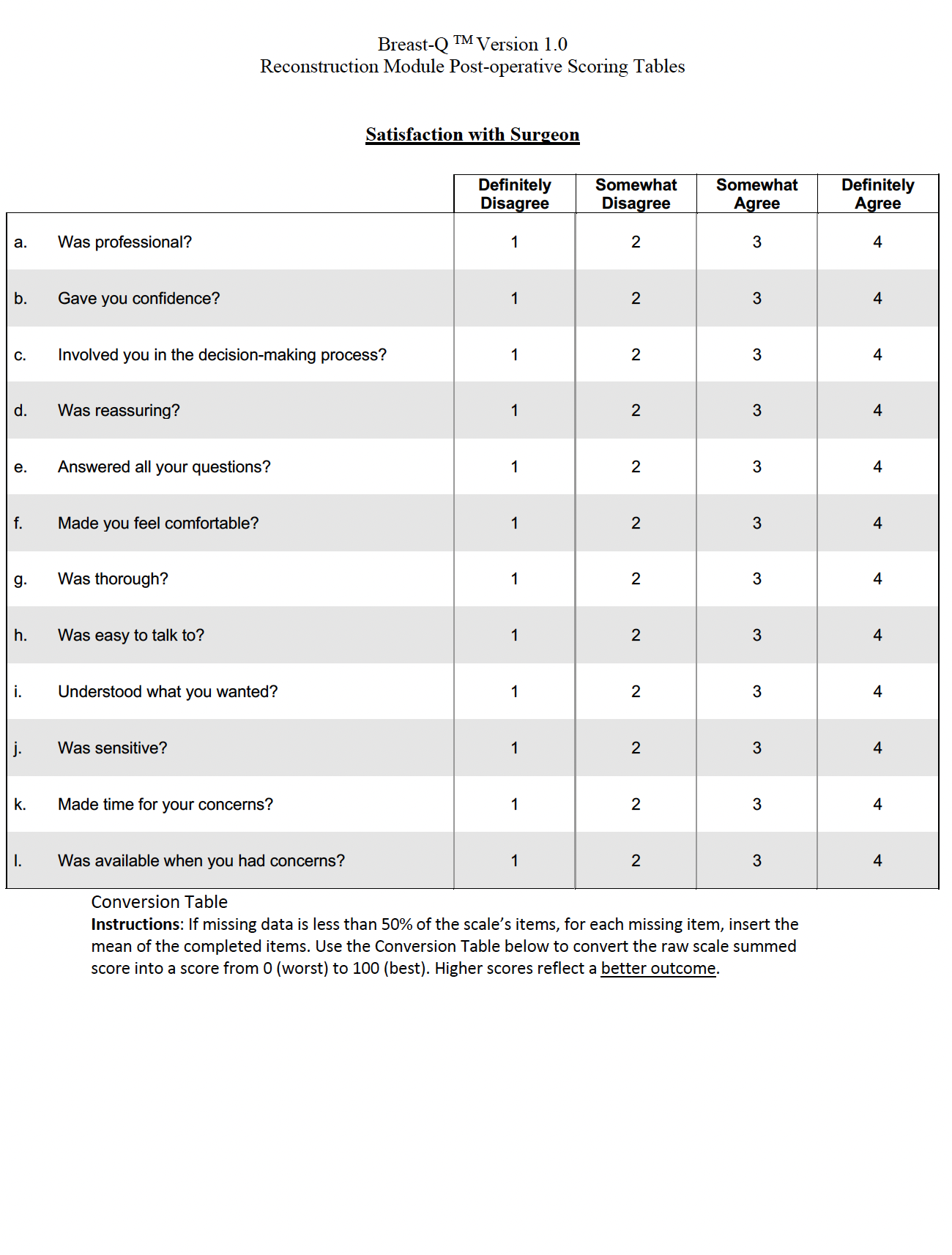


**
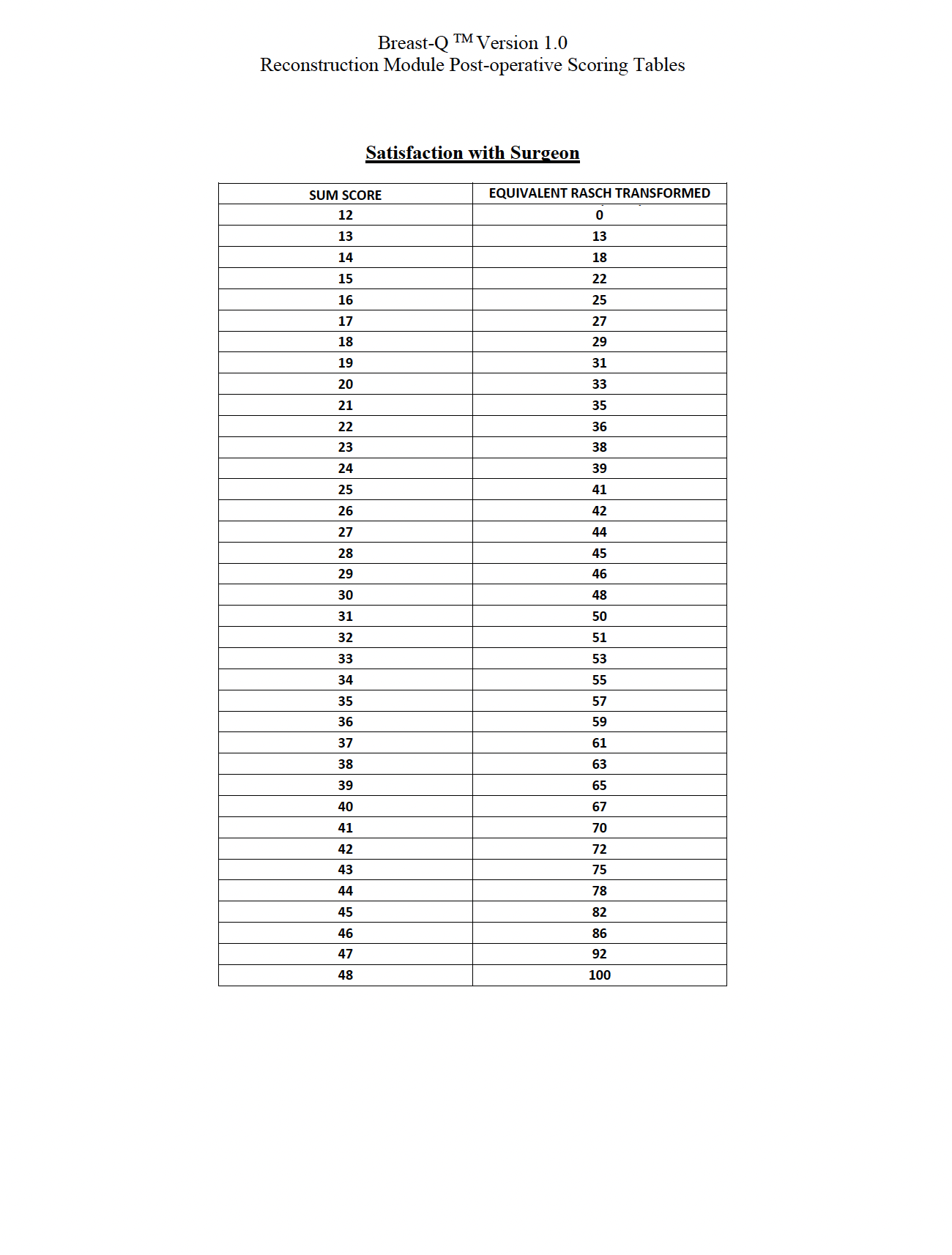
** **
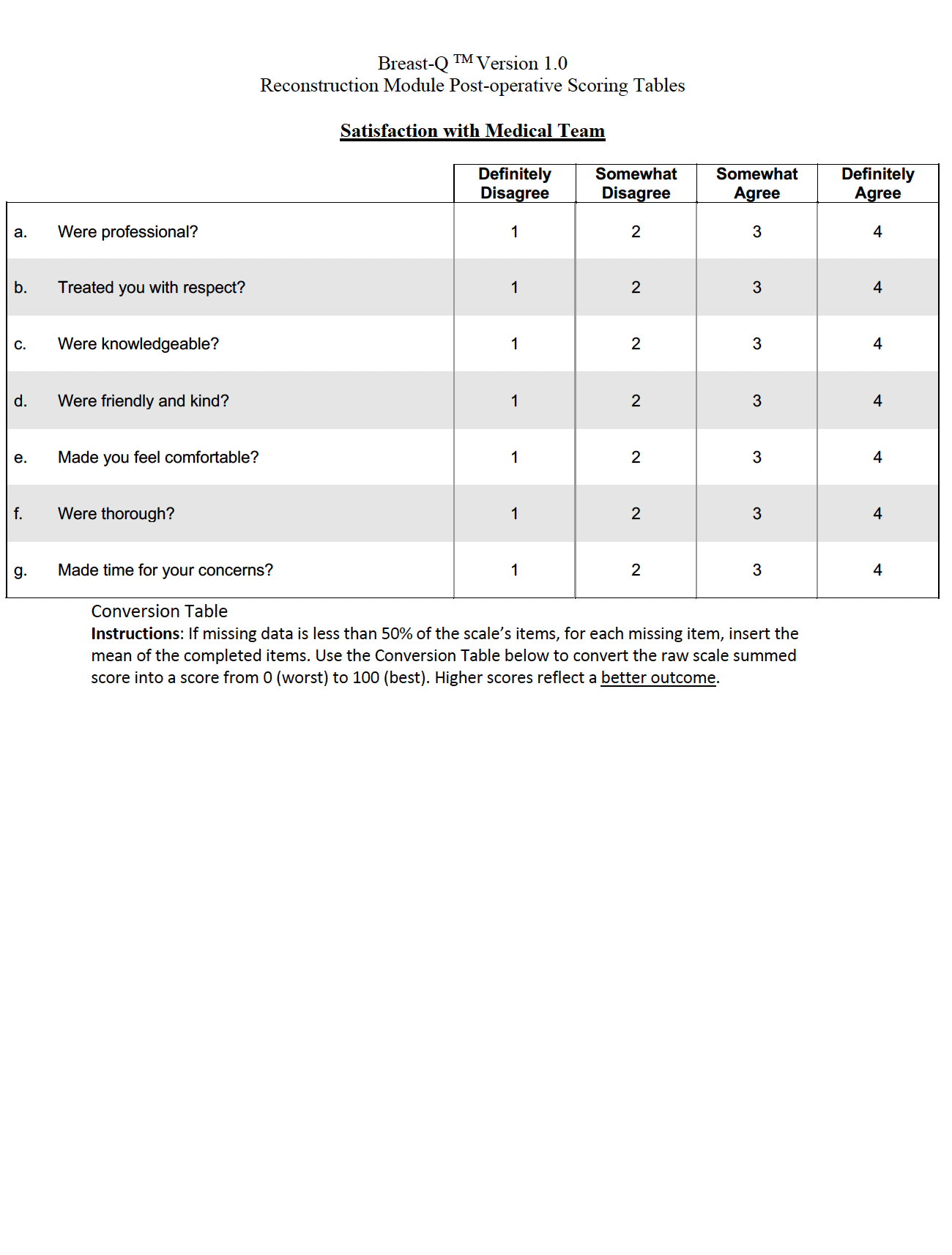
**
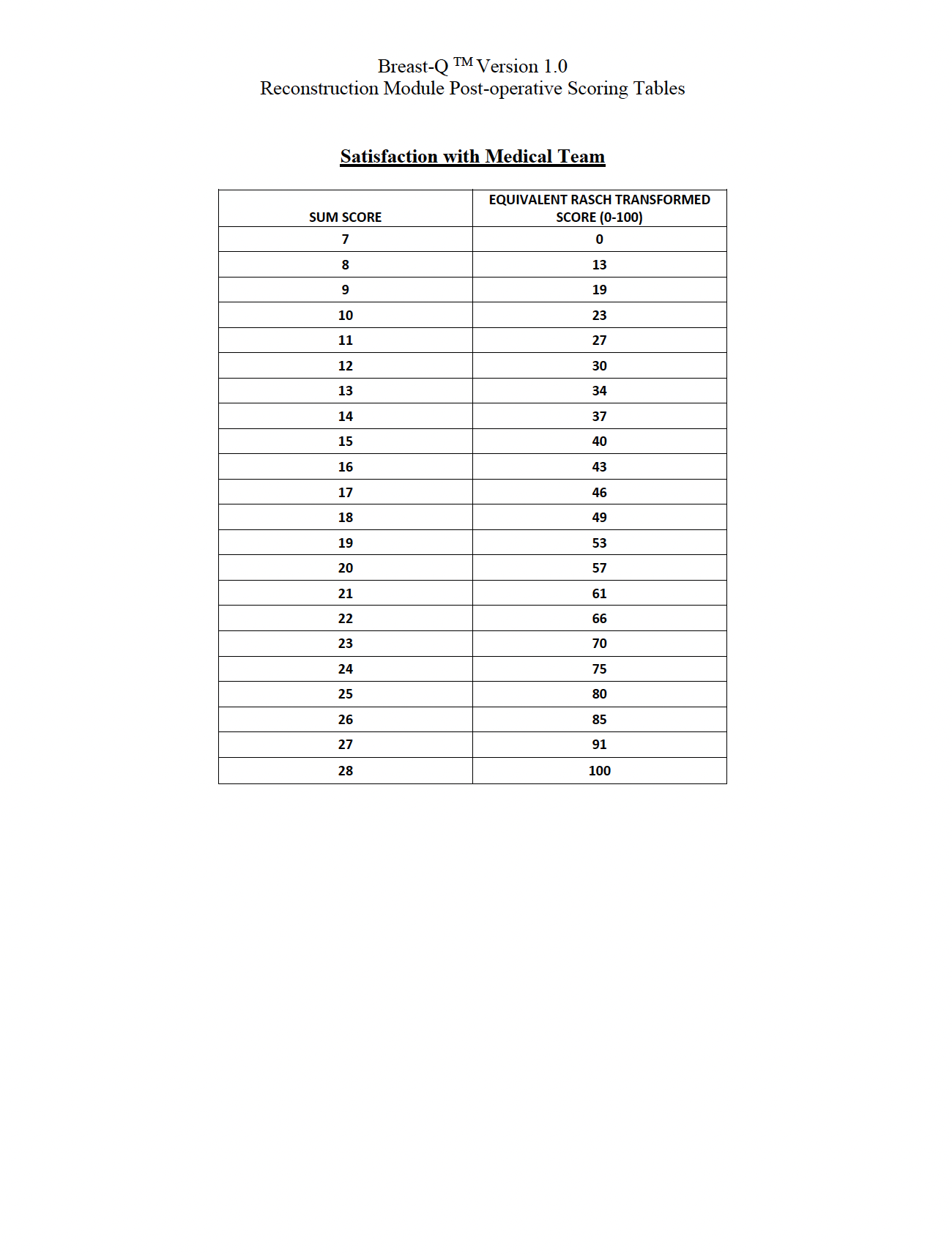

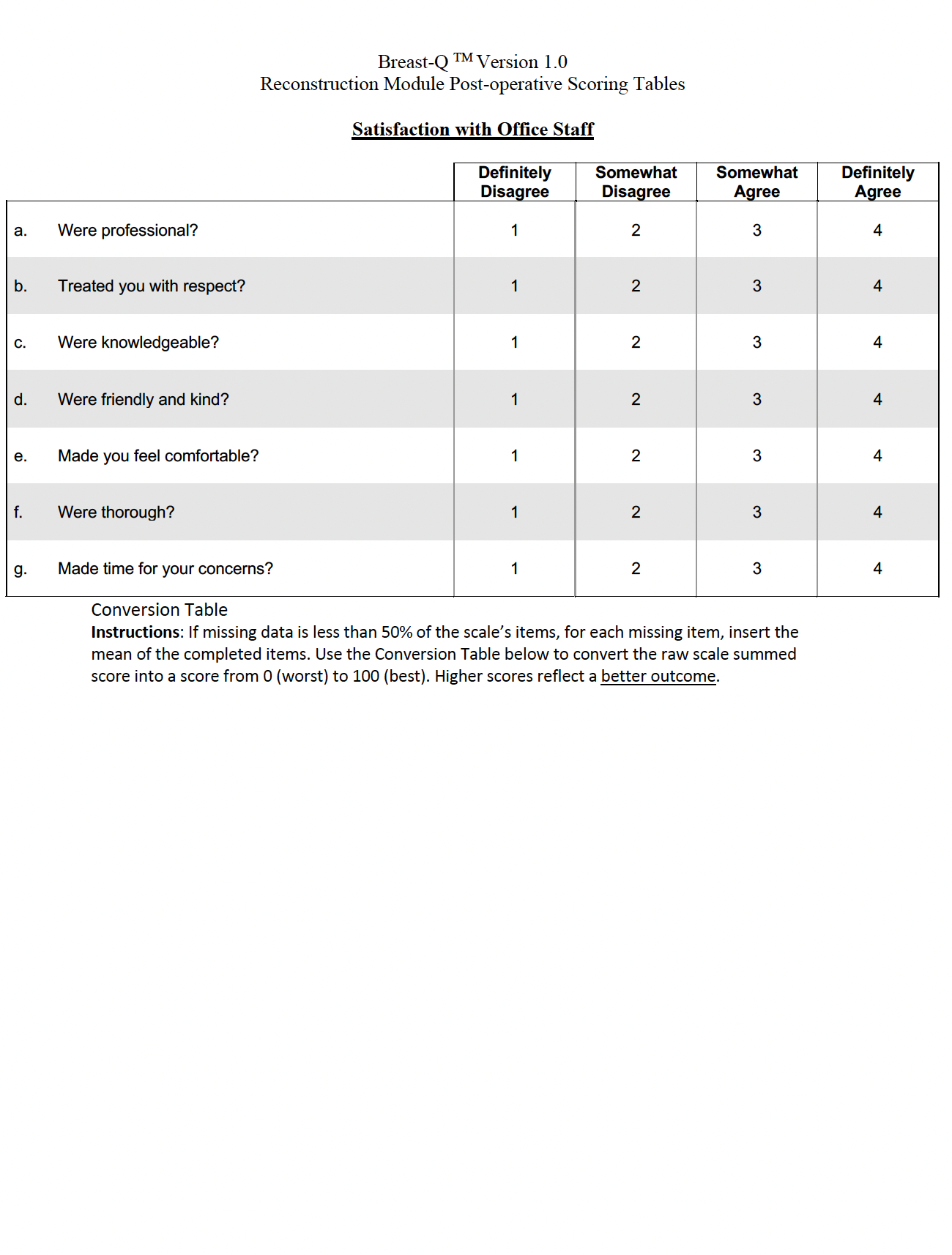

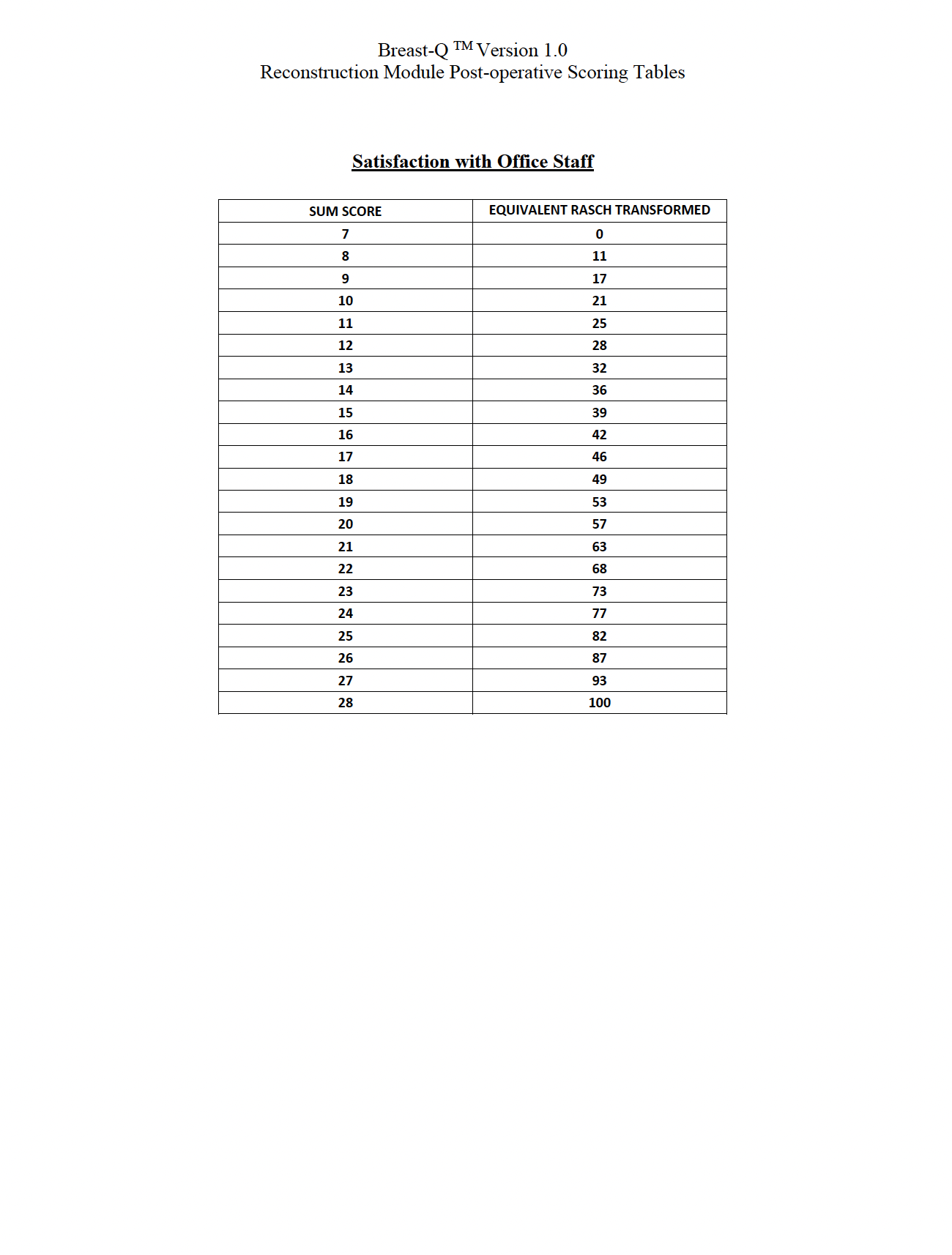

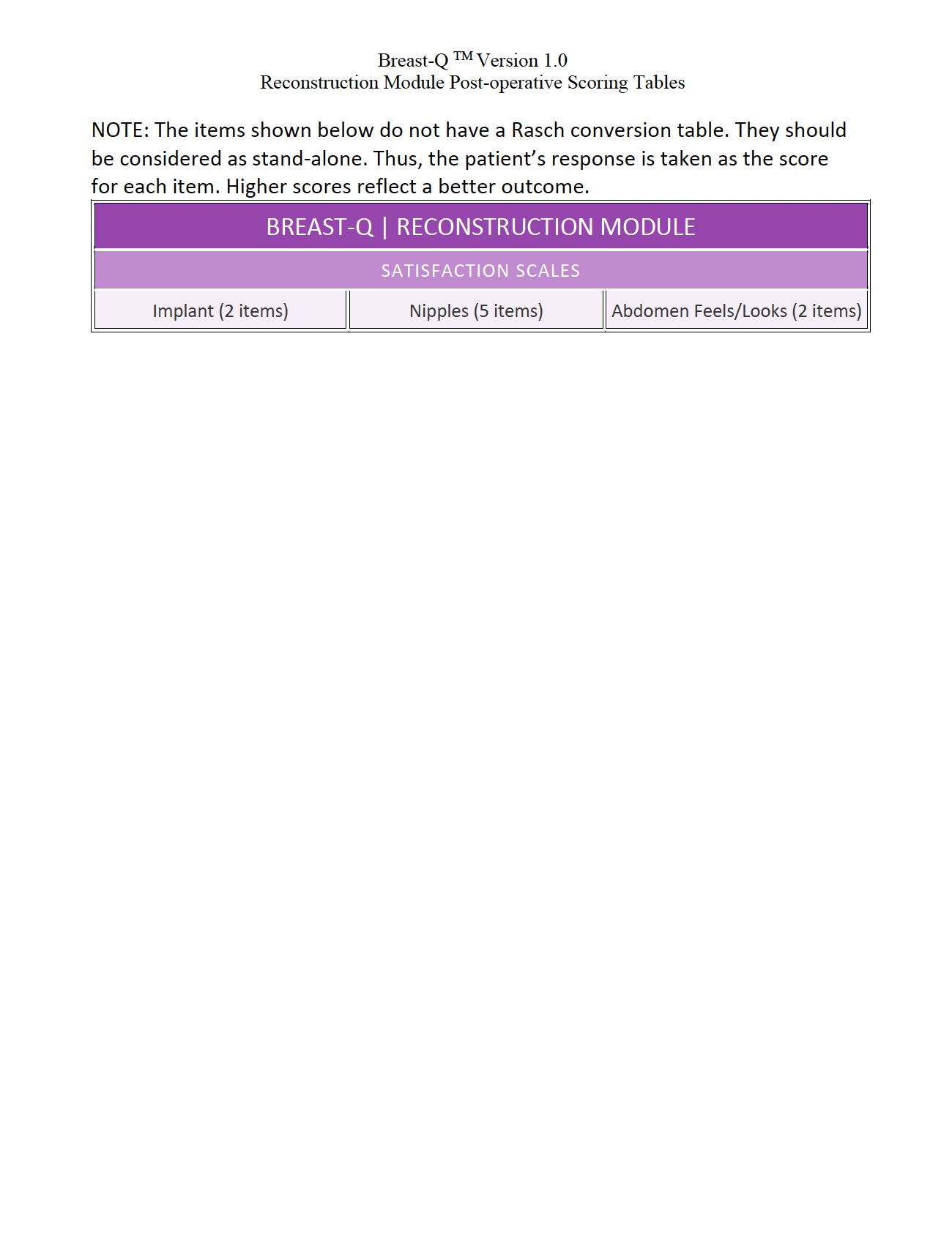

Supplement: zrag023_Supplementary_Data [file zrag023_supplementary_data.docx]
